# Supplementary material for: Production of probiotic garden cress (Lepidium Sativum) using Bifidobacterium Bifidum and its evaluation of nutritional value, biocontrol and growth rate ability
Source: PLoS One. 2025 Jun 4;20(6):e0322552. doi: 10.1371/journal.pone.0322552 (PMC12136354; doi:10.1371/journal.pone.0322552)
Supplement: S1 Table — (PDF) [file pone.0322552.s001.pdf]

S1 Table. Bacteria count (A), means (B) and analysis of variance (C)

A:

Day 0	Log form	Day 3	Log form
5E+13	13.6990	4E+11	11.6021
4E+13	13.6021	2E+11	11.3010
3E+13	13.4771	1.1E+11	11.0414

B:

Factor	N	Mean	StDev
Day 0	3	4.00000E+13	1.00000E+13
Day 3	3	2.36667E+11	1.48436E+11

Pooled StDev = 7.071847E+12

C:

F-Value	P-Value
47.42	0.002
