# Supplementary material for: Production of probiotic garden cress (Lepidium Sativum) using Bifidobacterium Bifidum and its evaluation of nutritional value, biocontrol and growth rate ability
Source: PLoS One. 2025 Jun 4;20(6):e0322552. doi: 10.1371/journal.pone.0322552 (PMC12136354; doi:10.1371/journal.pone.0322552)
Supplement: S2 Table — (PDF) [file pone.0322552.s002.pdf]

S2 Table. Acidity Measurement (A), means (B) and analysis of variance (C)

A:

Control	Treatment
0.013	0.016
0.009	0.019
0.015	0.020

B:

Factor	N	Mean	StDev
Control	3	0.01233	0.00306
Treatment	3	0.01833	0.00208

Pooled StDev = 0.00261406

C:

F-Value	P-Value
7.90	0.048
