# Supplementary material for: Production of probiotic garden cress (Lepidium Sativum) using Bifidobacterium Bifidum and its evaluation of nutritional value, biocontrol and growth rate ability
Source: PLoS One. 2025 Jun 4;20(6):e0322552. doi: 10.1371/journal.pone.0322552 (PMC12136354; doi:10.1371/journal.pone.0322552)
Supplement: S3 Table — (PDF) [file pone.0322552.s003.pdf]

**S3 Table. pH measurement of control and treatment samples (A), Means (B) and analysis of variance at minute one**

A:

| Control (minute 1) | Treatment (min 1) |
|---|---|
| 7.31 | 7.21 |
| 7.40 | 7.23 |
| 7.25 | 7.19 |

B:

| Factor | N | Mean | StDev |
|---|---|---|---|
| Control (min1) | 3 | 7.3200 | 0.0755 |
| Treatment (min 1) | 3 | 7.2100 | 0.0200 |

Pooled StDev = 0.0552268

C:

| F-Value | P-Value |
|---|---|
| 5.95 | 0.071 |
