# Supplementary material for: Production of probiotic garden cress (Lepidium Sativum) using Bifidobacterium Bifidum and its evaluation of nutritional value, biocontrol and growth rate ability
Source: PLoS One. 2025 Jun 4;20(6):e0322552. doi: 10.1371/journal.pone.0322552 (PMC12136354; doi:10.1371/journal.pone.0322552)
Supplement: S4 Table — (PDF) [file pone.0322552.s004.pdf]

S4 Table. pH measurement of control and treatment sample (A), means (B) and analysis of variance (C) at minute two

A:

Control (min 2)	Treatment (min 2)
7.13	6.59
7.16	6.61
7.01	6.39

B:

Factor	N	Mean	StDev
Control (min2)	3	7.1000	0.0794
Treatment (min 2)	3	6.5300	0.1217

Pooled StDev = 0.102713

C:

F-Value	P-Value
46.19	0.002
