# Supplementary material for: Production of probiotic garden cress (Lepidium Sativum) using Bifidobacterium Bifidum and its evaluation of nutritional value, biocontrol and growth rate ability
Source: PLoS One. 2025 Jun 4;20(6):e0322552. doi: 10.1371/journal.pone.0322552 (PMC12136354; doi:10.1371/journal.pone.0322552)
Supplement: S5 Table — (PDF) [file pone.0322552.s005.pdf]

S5 Table. Dry matter measurement (A), means (B) and analysis of variance (C)

Control	Treatment
94.01	94.10
94.06	94.25
94.08	94.37

B:

Factor	N	Mean	StDev
Control	3	94.0500	0.0361
Treatment	3	94.2400	0.1353

Pooled StDev = 0.0989949

C:

F-Value	P-Value
5.53	0.078
