# Supplementary material for: Production of probiotic garden cress (Lepidium Sativum) using Bifidobacterium Bifidum and its evaluation of nutritional value, biocontrol and growth rate ability
Source: PLoS One. 2025 Jun 4;20(6):e0322552. doi: 10.1371/journal.pone.0322552 (PMC12136354; doi:10.1371/journal.pone.0322552)
Supplement: S6 Table — (PDF) [file pone.0322552.s006.pdf]

S6 Table. Fat content measurement (A), means (B) and analysis of variance (C)

Control	Treatment
0.20	0.36
0.28	0.39
0.26	0.32

B:

Factor	N	Mean	StDev
Control	3	0.2467	0.0416
Treatment	3	0.3567	0.0351

Pooled StDev = 0.0385141

C:

F-Value	P-Value
12.24	0.025
