# Supplementary material for: Production of probiotic garden cress (Lepidium Sativum) using Bifidobacterium Bifidum and its evaluation of nutritional value, biocontrol and growth rate ability
Source: PLoS One. 2025 Jun 4;20(6):e0322552. doi: 10.1371/journal.pone.0322552 (PMC12136354; doi:10.1371/journal.pone.0322552)
Supplement: S7 Table — (PDF) [file pone.0322552.s007.pdf]

S7 Table. Colony count after gastrointestinal juice exposure (A), means of intestinal exposure results (B), means of colony count before gastrointestinal exposure (C), means of gastric juice exposure (D)

A:

Colony number before exposure	Gastric juice	Intestinal juice
4.00000E+11	6.40000E+10	1500000
2.00000E+11	7.40000E+10	4000000
1.00000E+11	5.10000E+10	3500000

B:

Variable	N	Mean	SE Mean	StDev
Intestinal juice	3	3000000	763763	1322876

C:

Variable	N	Mean	SE Mean	StDev
Colony count before exposure	3	2.33333E+11	8.81917E+10	1.52753E+11

D:

Variable	N	Mean	SE Mean	StDev
Gastric juice	3	6.30000E+10	6658328118	1.15326E+10
