# Supplementary material for: Production of probiotic garden cress (Lepidium Sativum) using Bifidobacterium Bifidum and its evaluation of nutritional value, biocontrol and growth rate ability
Source: PLoS One. 2025 Jun 4;20(6):e0322552. doi: 10.1371/journal.pone.0322552 (PMC12136354; doi:10.1371/journal.pone.0322552)
Supplement: S8 Table — (PDF) [file pone.0322552.s008.pdf]

S8 Table. Vitamin C content measurement (A), means (B), and analysis of variance (C)

A:

Control	Treatment
98.68	99.62
98.42	99.56
98.58	99.50

B:

Factor	N	Mean	StDev
Control	3	98.5600	0.1311
Treatment	3	99.5600	0.0600

Pooled StDev = 0.101980

C:

F-Value	P-Value
144.23	0.000
