# Supplementary material for: Production of probiotic garden cress (Lepidium Sativum) using Bifidobacterium Bifidum and its evaluation of nutritional value, biocontrol and growth rate ability
Source: PLoS One. 2025 Jun 4;20(6):e0322552. doi: 10.1371/journal.pone.0322552 (PMC12136354; doi:10.1371/journal.pone.0322552)
Supplement: S9 Table — (PDF) [file pone.0322552.s009.pdf]

S9 Table. Organoleptic properties scores of 15 panelists

Panelist 1 Scores

Row	Factors	Aroma					Taste					Texture					Color					Appearance				
	Scores	1	2	3	4	5	1	2	3	4	5	1	2	3	4	5	1	2	3	4	5	1	2	3	4	5
1	Control					✓				✓						✓				✓						✓
2	Treatment			✓							✓					✓					✓				✓	

Panelist 2 Scores

Row	Factors	Aroma					Taste					Texture					Color					Appearance				
	Scores	1	2	3	4	5	1	2	3	4	5	1	2	3	4	5	1	2	3	4	5	1	2	3	4	5
1	Control				✓						✓					✓					✓					✓
2	Treatment					✓				✓					✓						✓					✓

Panelist 3 Scores

Row	Factors	Aroma					Taste					Texture					Color					Appearance				
	Scores	1	2	3	4	5	1	2	3	4	5	1	2	3	4	5	1	2	3	4	5	1	2	3	4	5
1	Control			✓							✓					✓				✓						✓
2	Treatment	✓								✓						✓					✓				✓	

Panelist 4 Scores

Row	Factors	Aroma					Taste					Texture					Color					Appearance				
	Scores	1	2	3	4	5	1	2	3	4	5	1	2	3	4	5	1	2	3	4	5	1	2	3	4	5
1	Control					✓				✓					✓						✓					✓
2	Treatment					✓					✓					✓				✓						✓

Panelist 5 Scores

Row	Factors	Aroma					Taste					Texture					Color					Appearance				
	Scores	1	2	3	4	5	1	2	3	4	5	1	2	3	4	5	1	2	3	4	5	1	2	3	4	5
1	Control					✓					✓					✓				✓					✓	
2	Treatment				✓					✓					✓						✓				✓	

Panelist 6 Scores

Row	Factors	Aroma					Taste					Texture					Color					Appearance				
	Scores	1	2	3	4	5	1	2	3	4	5	1	2	3	4	5	1	2	3	4	5	1	2	3	4	5
1	Control				✓						✓					✓					✓					✓
2	Treatment					✓					✓				✓					✓						✓

Panelist 7 Scores

Row	Factors	**Aroma**					**Taste**					**Texture**					**Color**					**Appearance**				
	Scores	1	2	3	4	5	1	2	3	4	5	1	2	3	4	5	1	2	3	4	5	1	2	3	4	5
1	Control				✓					✓						✓					✓				✓	
2	Treatment					✓					✓					✓					✓					✓

Panelist 8 Scores

Row	Factors	**Aroma**					**Taste**					**Texture**					**Color**					**Appearance**				
	Scores	1	2	3	4	5	1	2	3	4	5	1	2	3	4	5	1	2	3	4	5	1	2	3	4	5
1	Control					✓					✓					✓					✓				✓	
2	Treatment				✓					✓						✓				✓					✓	

Panelist 9 Scores

Row	Factors	Aroma					Taste					Texture					Color					Appearance				
	Scores	1	2	3	4	5	1	2	3	4	5	1	2	3	4	5	1	2	3	4	5	1	2	3	4	5
1	Control				✓					✓						✓					✓					✓
2	Treatment				✓						✓					✓					✓					✓

Panelist 10 Scores

Row	Factors	Aroma					Taste					Texture					Color					Appearance				
	Scores	1	2	3	4	5	1	2	3	4	5	1	2	3	4	5	1	2	3	4	5	1	2	3	4	5
1	Control				✓						✓					✓					✓					✓
2	Treatment				✓						✓					✓					✓				✓	

Panelist 11 Scores

Row	Factors	**Aroma**					**Taste**					**Texture**					**Color**					**Appearance**				
	Scores	1	2	3	4	5	1	2	3	4	5	1	2	3	4	5	1	2	3	4	5	1	2	3	4	5
1	Control					✓					✓					✓					✓					✓
2	Treatment				✓					✓						✓				✓						✓

Panelist 12 Scores

Row	Factors	**Aroma**					**Taste**					**Texture**					**Color**					**Appearance**				
	Scores	1	2	3	4	5	1	2	3	4	5	1	2	3	4	5	1	2	3	4	5	1	2	3	4	5
1	Control			✓					✓							✓					✓					✓
2	Treatment	✓									✓					✓				✓						✓

Panelist 13 Scores

Row	Factors	Aroma					Taste					Texture					Color					Appearance				
	Scores	1	2	3	4	5	1	2	3	4	5	1	2	3	4	5	1	2	3	4	5	1	2	3	4	5
1	Control				✓				✓						✓						✓					✓
2	Treatment					✓					✓					✓					✓				✓	

Panelist 14 Scores

Row	Factors	Aroma					Taste					Texture					Color					Appearance				
	Scores	1	2	3	4	5	1	2	3	4	5	1	2	3	4	5	1	2	3	4	5	1	2	3	4	5
1	Control				✓					✓						✓					✓					✓
2	Treatment					✓					✓					✓				✓						✓

Panelist 15 Scores

Row	Factors	**Aroma**					**Taste**					**Texture**					**Color**					**Appearance**				
	Scores	1	2	3	4	5	1	2	3	4	5	1	2	3	4	5	1	2	3	4	5	1	2	3	4	5
1	Control					✓					✓				✓						✓					✓
2	Treatment					✓				✓						✓					✓					✓
