# Supplementary material for: Production of probiotic garden cress (Lepidium Sativum) using Bifidobacterium Bifidum and its evaluation of nutritional value, biocontrol and growth rate ability
Source: PLoS One. 2025 Jun 4;20(6):e0322552. doi: 10.1371/journal.pone.0322552 (PMC12136354; doi:10.1371/journal.pone.0322552)
Supplement: S10 Table — (PDF) [file pone.0322552.s010.pdf]

S10 Table. Aroma Analysis of variance (A) and means (B)

A:

F-Value	P-Value
0.45	0.506

B:

Factor	N	Mean	StDev
Control	15	4.267	0.704
Treatment	15	4.000	1.363

Pooled StDev = 1.08452
