# Supplementary material for: Production of probiotic garden cress (Lepidium Sativum) using Bifidobacterium Bifidum and its evaluation of nutritional value, biocontrol and growth rate ability
Source: PLoS One. 2025 Jun 4;20(6):e0322552. doi: 10.1371/journal.pone.0322552 (PMC12136354; doi:10.1371/journal.pone.0322552)
Supplement: S11 Table — (PDF) [file pone.0322552.s011.pdf]

S11 Table. Taste Analysis of Variance (A) and means (B)

A:

F-Value	P-Value
0.75	0.394

B:

Factor	N	Mean	StDev
Control	15	4.400	0.737
Treatment	15	4.600	0.507

Pooled StDev = 0.632456
