# Supplementary material for: Production of probiotic garden cress (Lepidium Sativum) using Bifidobacterium Bifidum and its evaluation of nutritional value, biocontrol and growth rate ability
Source: PLoS One. 2025 Jun 4;20(6):e0322552. doi: 10.1371/journal.pone.0322552 (PMC12136354; doi:10.1371/journal.pone.0322552)
Supplement: S14 Table — (PDF) [file pone.0322552.s014.pdf]

S14 Table. Appearance analysis of variance (A) and means (B)

A:

F-Value	P-Value
1.40	0.247

B:

Factor	N	Mean	StDev
Control	15	4.800	0.414
Treatment	15	4.600	0.507

Pooled StDev = 0.462910
