# Supplementary material for: Production of probiotic garden cress (Lepidium Sativum) using Bifidobacterium Bifidum and its evaluation of nutritional value, biocontrol and growth rate ability
Source: PLoS One. 2025 Jun 4;20(6):e0322552. doi: 10.1371/journal.pone.0322552 (PMC12136354; doi:10.1371/journal.pone.0322552)
Supplement: Attachment — Submitted filename: PONE-D-24-18993.pdf 9-6.pdf [file pone.0322552.s015.pdf]

PLOS ONE

Production of probiotic garden cress (Lepidium Sativum) using Bifidobacterium Bifidum and its evaluation of nutritional value, biocontrol and growth rate ability

--Manuscript Draft--

**Manuscript Number:**	PONE-D-24-18993
**Article Type:**	Research Article
**Full Title:**	Production of probiotic garden cress (Lepidium Sativum) using Bifidobacterium Bifidum and its evaluation of nutritional value, biocontrol and growth rate ability
**Short Title:**	Production of probiotic vegetable
**Corresponding Author:**	Golnaz Shambayati<br>University of Science and Culture<br>Tehran, Tehran IRAN, ISLAMIC REPUBLIC OF
**Keywords:**	Non-dairy probiotic, Garden cress, Bifidobacterium bifidum, bacterial inoculation, probiotic vegetable
**Abstract:**	Probiotics are one of the most beneficial elements in human health. Several studies have confirmed the health benefits of probiotics.<br>This research aimed to produce probiotic garden cress using Bifidobacterium bifidum and to investigate its nutritional value, biocontrol, and growth rate.<br>Bifidobacterium bifidum (bb12) was inoculated into sterile cress seeds and the probiotic plant culture was done on days 0 and 3 after harvesting, in a TOS medium containing MUP (Mupirisin antibiotic). The chemical properties (such as pH, acidity, and dry matter) of both samples were determined and the comparison of growth rate, fat content, vitamin C, and organoleptic properties was done. Bacterial survival in SGI conditions and its antifungal effect on Rhizoctonia solani were also analyzed.<br>The number of bacteria on days 0 and 3 was CFU/g and 2.3 CFU/g respectively, which confirms the probiotic nature of the product (bacteria). However, no significant difference was observed in the physicochemical parameters. Bacterial viability was reported as 6.3 CFU/g in simulated gastric condition and as CFU/g in simulated intestine condition, and the antifungal effect of Bifidobacterium on R. solani was 15%.
**Order of Authors:**	Golnaz Shambayati<br>Mojtaba Mohammadzadeh Vazifeh<br>Seyed Masoud Hosseini<br>Hasan Hajjami Barkousaraei
**Additional Information:**	
**Question**	**Response**
**Financial Disclosure**<br><br>Enter a financial disclosure statement that describes the sources of funding for the work included in this submission. Review the submission guidelines for detailed requirements. View published research articles from *PLOS ONE* for specific examples.<br><br>This statement is required for submission and **will appear in the published article** if the submission is accepted. Please make sure it is accurate.	The author(s) received no specific funding for this work.

Powered by Editorial Manager® and ProduXion Manager® from Aries Systems Corporation

**Funded studies**<br>Enter a statement with the following details:<br>• Initials of the authors who received each award<br>• Grant numbers awarded to each author<br>• The full name of each funder<br>• URL of each funder website<br>• Did the sponsors or funders play any role in the study design, data collection and analysis, decision to publish, or preparation of the manuscript?<br><br>Did you receive funding for this work?	
**Competing Interests**<br><br>Use the instructions below to enter a competing interest statement for this submission. On behalf of all authors, disclose any competing interests that could be perceived to bias this work—acknowledging all financial support and any other relevant financial or non-financial competing interests.<br><br>This statement is required for submission and **will appear in the published article** if the submission is accepted. Please make sure it is accurate and that any funding sources listed in your Funding Information later in the submission form are also declared in your Financial Disclosure statement.<br><br>View published research articles from *PLOS ONE* for specific examples.	The authors have declared that no competing interests exist.

**NO authors have competing interests**<br><br>Enter: *The authors have declared that no competing interests exist.*<br><br>**Authors with competing interests**<br><br>Enter competing interest details beginning with this statement:<br><br>*I have read the journal's policy and the authors of this manuscript have the following competing interests: [insert competing interests here]*<br><br>* typeset	
**Ethics Statement**<br><br>Enter an ethics statement for this submission. This statement is required if the study involved:<br><br>• Human participants<br>• Human specimens or tissue<br>• Vertebrate animals or cephalopods<br>• Vertebrate embryos or tissues<br>• Field research<br><br>Write "N/A" if the submission does not require an ethics statement.<br><br>General guidance is provided below. Consult the submission guidelines for detailed instructions. **Make sure that all information entered here is included in the Methods section of the manuscript.**	N/A

**Format for specific study types**<br><br>**Human Subject Research (involving human participants and/or tissue)**<br>• Give the name of the institutional review board or ethics committee that approved the study<br>• Include the approval number and/or a statement indicating approval of this research<br>• Indicate the form of consent obtained (written/oral) or the reason that consent was not obtained (e.g. the data were analyzed anonymously)<br><br>**Animal Research (involving vertebrate animals, embryos or tissues)**<br>• Provide the name of the Institutional Animal Care and Use Committee (IACUC) or other relevant ethics board that reviewed the study protocol, and indicate whether they approved this research or granted a formal waiver of ethical approval<br>• Include an approval number if one was obtained<br>• If the study involved *non-human primates*, add *additional details* about animal welfare and steps taken to ameliorate suffering<br>• If anesthesia, euthanasia, or any kind of animal sacrifice is part of the study, include briefly which substances and/or methods were applied<br><br>**Field Research**<br><br>Include the following details if this study involves the collection of plant, animal, or other materials from a natural setting:<br>• Field permit number<br>• Name of the institution or relevant body that granted permission	
**Data Availability**<br><br>Authors are required to make all data underlying the findings described fully available, without restriction, and from the time of publication. PLOS allows rare exceptions to address legal and ethical concerns. See the PLOS Data Policy and FAQ for detailed information.	Yes - all data are fully available without restriction

A Data Availability Statement describing where the data can be found is required at submission. Your answers to this question constitute the Data Availability Statement and **will be published in the article**, if accepted.<br><br>**Important:** Stating 'data available on request from the author' is not sufficient. If your data are only available upon request, select 'No' for the first question and explain your exceptional situation in the text box.<br><br>Do the authors confirm that all data underlying the findings described in their manuscript are fully available without restriction?	
**Describe where the data may be found in full sentences. If you are copying our sample text, replace any instances of XXX with the appropriate details.**<br><br>• If the data are **held or will be held in a public repository**, include URLs, accession numbers or DOIs. If this information will only be available after acceptance, indicate this by ticking the box below. For example: *All XXX files are available from the XXX database (accession number(s) XXX, XXX.*).<br>• If the data are all contained **within the manuscript and/or Supporting Information files**, enter the following: *All relevant data are within the manuscript and its Supporting Information files.*<br>• If neither of these applies but you are able to provide **details of access elsewhere**, with or without limitations, please do so. For example:<br><br>*Data cannot be shared publicly because of [XXX]. Data are available from the XXX Institutional Data Access / Ethics Committee (contact via XXX) for researchers who meet the criteria for access to confidential data.*<br><br>*The data underlying the results presented in the study are available from (include the name of the third party*	The data underlying the results presented in the study are available from (Golnaz Shambayati/gshambayati97@gmail.com).

and contact information or URL).<br>• This text is appropriate if the data are owned by a third party and authors do not have permission to share the data.<br><br>* typeset	
Additional data availability information:	Tick here if the URLs/accession numbers/DOIs will be available only after acceptance of the manuscript for publication so that we can ensure their inclusion before publication.

Powered by Editorial Manager® and ProduXion Manager® from Aries Systems Corporation

Manuscript Click here to access/download;Manuscript;Final (1) - Copy.docx

Title

Production of probiotic garden cress (*Lepidium Sativum*) using *Bifidobacterium Bifidum* and its evaluation of nutritional value, biocontrol and growth rate ability

Golnaz Shambayati[1], Mojtaba Mohammadzadeh Vazifeh[1*], Seyed Masoud Hosseini[2], Hasan Hajjami Barkousaraei[1]

[1] Faculty of Modern Biological Sciences and Technologies, University of Science and Culture, Tehran, Iran

[2] Faculty of Biological Sciences and New Technologies, SBU, Tehran, Iran

***Corresponding Author:**

Mojtaba Mohammadzadeh Vazifeh

Email: mohammadzadeh.vazifeh@usc.ac.ir

Abstract

Probiotics are one of the most beneficial elements in human health. Several studies have confirmed the health benefits of probiotics.

This research aimed to produce probiotic garden cress using *Bifidobacterium bifidum* and to investigate its nutritional value, biocontrol, and growth rate.

Bifidobacterium bifidum (bb12) was inoculated into sterile cress seeds and the probiotic plant culture was done on days 0 and 3 after harvesting, in a TOS medium containing MUP (Mupirisin antibiotic). The chemical properties (such as pH, acidity, and dry matter) of both samples were determined and the comparison of growth rate, fat content, vitamin C, and organoleptic properties was done. Bacterial survival in SGI conditions and its antifungal effect on *Rhizoctonia solani* were also analyzed.

The number of bacteria on days 0 and 3 was 4×10^{13} CFU/g and 2.3×10^{11} CFU/g respectively, which confirms the probiotic nature of the product ($>10^{6}$ bacteria). However, no significant difference was observed in the physicochemical parameters. Bacterial viability was reported as 6.3×10^{10} CFU/g in simulated gastric condition and as 3×10^{6} CFU/g in simulated intestine condition, and the antifungal effect of *Bifidobacterium* on *R. solani* was 15%.

Abbreviation:

SGI: simulated gastrointestinal

Introduction

The gut microbiota is mostly shaped by the environment, in particular the diet. Probiotics are defined as living microorganisms that affect the health of the host by improving the composition of gut microflora [1]. Consumption of probiotics has beneficial effects on the prevention of various diseases [2]. It is commonly accepted that probiotics must be present at a minimum level, ranging from 10^6-10^9 CFU/ml of the product, to be beneficial [3].

Common microorganisms used in probiotic production are predominantly *Lactobacillus*, and *Bifidobacterium* species which produce organic acid and lower the intestinal pH [4]. Probiotic strains should be resistant to gastrointestinal conditions, and be safe for human use [5].

Most probiotic foods are milk-based but certain sectors of the population such as those allergic to milk proteins, those who are lactose intolerant, and those who are vegetarian, cannot consume dairy products. Therefore, a need has arisen among consumers for an alternative to fermented dairy products by exploring new non-dairy matrices as probiotics carriers [6], [7]. Fruit and vegetables are nondairy origin matrices and have great potential to preserve probiotic viability either due to their morphology or because they act as substrates or prebiotics [2].

Lepidium sativum, also known as garden cress, is a fast-growing annual herbaceous plant belonging to the *Brassicaceae* family [8]. It is used in the treatment of cough and bleeding piles. Leaves are mildly stimulant, diuretic, and useful in scorbutic diseases and liver complaints [9].

In this research, we tried to produce a probiotic garden cress by inoculating its seeds with *Bifidobacterium bifidum* (bb12) bacteria.

Materials and methods

Materials

Probiotic cells, *Bifidobacterium bifidium* (bb12), were provided by (Chr-Hansen A/S., Hørsholm, Denmark) and *Rhizoctonia*. *solani* fungi (IBRC-M 30523) was provided to determine the antifungal activity of *Bifidobacterium*. MRS Broth and MRS Agar mediums (Ibresco Co., Iran) and TOS Agar (Biomark, India) were used for culture activation and *Bifidobacterium* growth. PDA Agar (Merck, Kenilworth, NJ, USA) was used as the main medium for *Rhizoctonia*. *solani* culture. Hydrogen chloride (HCl), Sodium hydroxide (NaOH), and n-hexane solutions were purchased from (Dr. Mojallali Industrial Chemical Co., Tehran, Iran). Trypsin (Sigma Aldrich Co., St. Louis, MO, USA), Ox gall (Sigma Aldrich) and Pepsin (Sigma Aldrich) were utilized to prepare simulated gastrointestinal conditions.

Production of probiotic garden cress

Culture revival

The MRS broth medium was sterilized in an autoclave at 121℃ for 15 minutes. Bacteria were cultured in a sterile MRS broth medium and incubated anaerobically at 37℃ for 48-72 hours [10].

Garden cress seed preparation

Surface sterilization

100 grams of fresh garden cress seeds were purchased from the grocery store and divided equally into two sterile beakers. The seeds were surface sterilized in 70% ethanol for 1 minute, and hypochlorite solution for 10 minutes. Finally, the seeds were rinsed 5 times with sterile distilled water [11].

Seed mucilage generation

Each seed sample (50 g) was soaked separately in two containers (each containing 0.5 liters of sterile distilled water) for 12 hours [12].

Seed inoculation

The medium suspension containing *Bifidobacterium* strains was separated by centrifugation (Hettich Instruments, Tuttlingen, Germany) at 4500 rpm for 10 minutes at room temperature and after being washed twice with PBS buffer (Merck), the bacterial sediment was separated [13]. 50 g of seeds were mixed with the inoculant containing *Bifidobacterium bifidum* at a ratio of 1:100 (inoculant: seed weight). The total volume of the inoculant was 24 ml and its turbidity was equal to 0.5 McFarland (1.5×10^{8} CFU/ml) (Fig 1). The seeds were kept in the inoculum for 2 hours [14].

Seed planting

6 vases (3 vases for each treatment) and cocopeat soil were prepared for planting seeds. 10 kg of soil was added to each vase [15]. 2 liters of water were added to each vase to mois5ten the soil [16]. The plants were watered with 0.5 liters of water twice a day [17].

Plant harvesting

Both treatments were harvested one week after planting when the plant height reached 5 cm. Both samples were separately transferred to the laboratory in sterile plastics [18].

Comparison of duration and rate of growth

This was done by preparing a table and checking the height of the plant daily with a caliper.

Bacterial enumeration

Bacterial enumeration was done according to the method described by Arepally and Goswami [10] with slight modifications. Five grams of the bacteria-containing sample were washed, cut, and dissolved in a 45 ml PBS solution and the serial dilution was performed. 1 ml of each dilution was cultured in TOS medium containing MUP (Merk) supplement by pour plate method and incubated in an anaerobic jar (Afshar Co., Iran) at 37℃ for 72 hours. The viability of bacteria was examined on days 0 and 3. Samples were kept from day 0 to 3 at refrigerator temperature (4℃) in plastic bags.

Physicochemical measurements

pH

The pH of both samples was measured according to OECD guidelines for the testing of chemicals. After calibrating the pH meter (Crison Co., France), 5 grams of sample was mixed with 50 milliliters of distilled water, and the pH value was read at minutes 1 and 2 [19].

Acidity

The acidity of both samples was measured according to OECD guidelines for the testing of chemicals. To determine the acidity, 5 grams of each sample was titrated with 0.1N sodium hydroxide. The acidity of both samples was measured using the following formula:

Acidity (calculated as linoleic acid) = 1.31 $t \times c_1 / w$ (%)

Where;

c_1 = (NaOH). mol/L (normality) of the solution

t = volume (mL) of NaOH solution

w = weight (g)of a sample [19]

Dry matter percentage measurement

Dry matter was determined by drying the weighed samples in an oven (Shimaz, Iran) at 78℃ for 24h (Fig 2). the following formula was used to measure the percentage of dry matter [20], [21]:

Percentage of dry matter = fresh sample weight-dry sample weight/fresh sample weight ×100

Viability of bacteria under simulated gastrointestinal conditions

Simulated gastric juice was prepared by adding 0.35 g of pepsin in 100 ml of 0.2% saline and adjusting the pH to 2.0 and 2.5 with hydrochloric acid. Simulated intestinal juice was prepared by adding 0.1 g of trypsin and 1.8 g of bile salts in a sterile solution of 1.1 g of sodium bicarbonate and 0.2 g of sodium chloride in 100 ml distilled water and adjusting pH to 8.0 with 0.5 M sodium hydroxide (Fig 3). Both solutions were sterilized with filtering through 0.45 µm filter [22].

Determination of total viable count

The changes in the total number of living bacteria during the treatment with gastric juice (3 hours) and intestinal juice (3 hours) and were determined by the pour plate method using MRS agar on day 3. The plates were incubated anaerobically at 37℃ for 48 hours. The number of *Bifidobacterium bifidum* in samples before and after the treatment was counted and compared. The viability of the bacteria was calculated according to the following equation:

Survival rate (%) = $\log \mathrm{CFU}\ N_1 / \log \mathrm{CFU}\ N_0 \times 100$

Where N_1 represents the total viable count of *Bifidobacterium bifidum* after treatment by simulated gastrointestinal juices, and N_0 represents the total viable count of *Bifidobacterium bifidum* before treatment [22].

Fat content

Fat content determination was done according to the method described by Stefano et al. [23] with slight modifications. Three grams of each sample were extracted in n-hexane solvent using a Soxhlet extractor for 6 hours. The extract was filtered and concentrated in a rotary evaporator at 40℃. The amount of extracted oil was obtained using the following formula:

Fat percentage = fat weight/sample weight ×100

Vitamin C determination

The amount of vitamin C was determined according to ISO 6557/2-1984: Fruits, vegetables and derived products - Determination of ascorbic acid - Part 2: Routine methods [24]. The amount of vitamin C was measured by extracting ascorbic acid from the samples, using the oxalic acid (Sigma Aldrich) solution with the acetic acid, and titration with 2,6-dichlorophenol-indophenol (Merk) until a light pink color appeared (Fig 4). Vitamin C content (mg) was calculated according to the following equation:

Vitamin C = $(V_0 - V_1) \times m_1 / m_0 \times 100$

Where V_0 represents the volume of dye solution (ml) used for titration, V_1 represents the volume of the dye solution (ml) used in the control test, m_1 represents the mass

of ascorbic acid (mg) and m_0 represents the mass of the sample (g) used in the titration test.

Sensory evaluation

The characteristics of Garden cress in terms of taste (spiciness), aroma, texture, color, and appearance were examined by five panelists. The organoleptic evaluation in this research was based on the hedonic sensory evaluation, which is a 5-point method (from most unfavorable to most favorable). Testing was carried out by giving a questionnaire to the panelists with parameters, i.e., color, aroma, taste, texture, and appearance. To achieve quantitative results, the qualitative results obtained from the test were converted into quantitative (parametric) data and results by giving the points from 1 to 5 to properties [25].

Antifungal activity of *Bifidobacterium bifidum*

The agar well diffusion method was used to analyze the antifungal activity of bacteria. To prepare the supernatant, *Bifidobacterium bifidum* bacteria were cultured on an MRS broth medium for 48 hours. Then it was centrifuged at 3000 rpm for 15 minutes. 1 cc of the supernatant was filtered by a 0.45 µm syringe filter. On the other hand, a plate containing sterile PDA agar medium was prepared and 4 wells with a diameter of 0.6 mm were bored using a sterile punch. 100 µl of the supernatant was added to the wells (until completely filled) and one well was considered as a control sample containing sterile distilled water. An agar plug (0.5×0.5 cm) was removed from the fresh culture of *Rhizoctonia solani*, and placed in the center of the plate and between the wells. The plate was incubated for 4 days at 25℃. The radius of the growth zone (cm) was measured in both samples, and the results were recorded after 4 days. Antifungal activity was calculated according to the following formula:

$$FI(\%) = R_c\text{-}R_t/R_c \times 100$$

Where R_c represents the radius of the growth zone in the control and R_t represents the radius of the growth zone in *Bifidobacterium*-containing treatment [26].

Results

Comparison of duration and rate of growth

The result of this measurement and comparison of the data showed that the addition of *Bifidobacterium* to the plant did not change the rate and time of its growth (Fig

5), and both treatments had equal growth under the same planting conditions in terms of light and watering (Fig 6).

Probiotic count

The number of *bifidobacteria* in the garden cress was determined as the average of three counts (Fig 7 and 8). According to recommendations, there should be a minimum of 10^6 CFU/ml or g of live probiotic bacteria at the time of consumption. Counting *bifidobacteria* in inoculated garden cress shows that plant is indeed a probiotic vegetable.

Physicochemical properties analysis

The pH value of the control sample and the *bifidobacteria* – containing one was determined at minutes one and two (Fig 9). The difference in the pH of the two treatments and the low pH of the probiotic sample can confirm the presence of *Bifidobacterium* (which produces acid) in the garden cress (Fig 10).

To determine the acidity, 5 grams of each sample was measured, and individually titrated with 0.1N sodium hydroxide. The volume of sodium hydroxide used for control and probiotic samples was 0.55 ml and 0.66 ml, respectively (Fig 11). The acidity of each sample was calculated as follows:

Control= $1.31 \times 0.1 \times 0.55/5 = 0.014\%$

Probiotic= $1.31 \times 0.1 \times 0.66/5 = 0.017\%$

Dry matter

The weight of the samples before and after being placed in the oven was measured and the percentage of dry matter was calculated according to the below formula:

Control sample = $3.330\text{-}0.198/3.330 \times 100 = 94\%$

Probiotic sample = $3.320\text{-}0.191/3.320 \times 100 = 94\%$

The viability of *bifidobacteria* under gastrointestinal conditions

The number of viable *bifidobacteria* in the garden cress reached 6.3×10^{11} CFU/g after exposure to the simulated gastric condition. The number of viable *bifidobacteria* was 3×10^6 CFU/g under simulated intestinal conditions. So, we conclude that the resulting product could survive in the simulated gastrointestinal conditions, which is a significant point because one of the points in the production of probiotic foods is their survival in the human digestive system and resistance to gastric and intestinal conditions.

Fat content

The amount of fat (%) was determined by measuring the mass of the samples and the Soxhlet extraction apparatus before and after being placed in a Soxhlet extractor and rotary evaporator.

The results of this measurement show that the presence of *Bifidobacterium* did not change the amount of fat in garden cress and the percentage of fat in both control and probiotic samples was almost the same (Fig 12).

Vitamin C content

The volume of dye solution used for titration was 0.2 ml, the volume of the solution used in the control test was 0.1 ml, the mass of the ascorbic acid was 50 mg, and the mass of the samples used for the titration was approximately 5 grams (Fig 13). the results show no significant difference and both samples have equal amount of Vitamin C.

Organoleptic properties analysis

Five panelists were done the organoleptic evaluation of both control and probiotic samples, the results of which are shown as the average of each parameter (from 1 to 5) in the graph below (Fig 14):

Antifungal activity of *Bifidobacterium bifidum* (bb12)

The radius of the growth zone in the control sample was reported as 3.8 cm, and in the sample containing *Bifidobacterium*, it was reported as 3.2 cm. The percentage of fungi growth inhibition by *Bifidobacterium* was calculated according to the following formula:

FI = $3.8\text{-}3.2/3.8 \times 100 = 15\%$

Discussion

In confirmation of the obtained results in the enumeration and survival of bacteria and the decrease in the number of them during the storage period, Babak Pakbin et al. [27] observed that the number of *Lactobacillus delbruecki* bacteria in their product reached approximately 10×10^9 CFU/ml and it was suitable for consumption as a probiotic drink. A decrease in the number of bacteria was observed from weeks 0 to 4, but there was no change in the probiotic properties of the product. Tatiana Colombo Pimentel et al. [28] investigated the probiotic viability of apple juice supplemented with two probiotic strains and reported a rapid loss in the number of bacteria during the storage of juices. It has been reported by Min et al. [29] that the food matrix used to inoculate probiotic strains can affect their viability and announced that the population of the three strains used, reduced with increasing storage time. Ankita Kataria et al. [30] reported that it is possible to deliver

Bifidobacterium longum through ice cream. The legume sprouts and *Lb. plantarum* 299V can be successfully combined to form a new probiotic-rich food [31]. The enumeration of the bacteria in the present study showed the essential count for probiotic bacteria to exhibit beneficial effects.

We observed a lower pH and higher acidity in the probiotic sample in comparison with the control one, which confirms the presence of *Bifidobacterium* and its acid production but no changes in dry matter were reported. Oliveira et al. [32] reported an increase in dry matter of goat cheese and stated that this could be related to an increase in the percentage of protein. So, we can conclude that changes in dry matter might be observed in dairy probiotic products. Vinderola et al. [33] reported that the pH values in two types of yogurt containing *Bifidobacterium* and *Lactobacillus* were negligible during the refrigerated storage. The garden cress pH in the present study was in the range of 6.5-7.2 which includes the optimum pH for the growth of *Bifidobacterium*. Many research has confirmed that the presence of probiotic strains in products does not have a significant effect on the titratable acidity or may increase it [34]. The decrease in pH value in honey yogurt containing *Lactobacillus reuteri* DPC16 during the 3 weeks of storage was also reported by Anand Mohan et al. [35].

The survival of probiotic strains is an important factor for exertion their health benefits. In present study, the viability of *Bifidobacterium* decreased after exposure to SGI condition but still was higher than the minimal range accepted to be beneficial ($\geq 10^6$ CFU/ml or g). This result is similar to those reported by Casarotti et al. [36] who demonstrated that the viability of probiotic strains significantly decreased after exposure to in vitro gastrointestinal conditions. Moreover, Chávarri et al. [37] observed a rapid loss in the number of free bacterial cells in SGI conditions. In a more recent study by Khorasani and Shojaosadati [38] it has been found that the number of viable probiotic bacteria decreased during exposure to SGI conditions.

It has been demonstrated by many studies that the fat content of foods provides protection for probiotic bacteria. In the present study, the fat content of probiotic sample was not significantly higher than the control one. Ciesielska et al. [17] have shown that the kind of water is what affects the fat content in garden cress. Popova [39] reported that the addition of probiotic strains reduces the saturated fatty acids and increases the polyunsaturated fatty acids of meat, and thus has a positive effect on it.

We also observed that the Vitamin C content was not affected by *Bifidobacterium* and its value difference in both samples was 1 mg (higher in probiotic sample). AdebayoTayo et al. [25] found that the vitamin C content in probiotic pineapple juice had no significant differences during storage but no significant change in Vitamin C content of the fermented Acerolla ice cream was observed by Favaro Tritade et al. [40]. Flores-Félix et al. [41] reported that the vitamin C highly increased in the strawberries inoculated with plant probiotics.

The fungal growth inhibition of *Bifidobacterium* against *R. solani* that we observed in present study was 15%. Xin Ma et al. [42] also worked on another probiotic strain, *Bacillus subtilis*, and reported that *B. subtilis* has been used widely in agriculture as a biocontrol agent and has a high antifungal activity against *R. solani*. Gerbaldo et al. [43] have also indicated the antifungal effect of two *Lactobacillus* strains against aflatoxigenic fungal isolate.
The organoleptic evaluation showed no difference in *Bifidobacterium* – containing garden cress. Coman et al. [44] worked on four probiotic foods, and reported that no significant differences were observed between probiotic-enriched foods and control foods. Afzaal et al. [45] demonstrated that the taste and the flavor of probiotic and control ice cream were the same and no difference was observed.

Conclusion

In this study, we produced probiotic garden cress by inoculating *Bifidobacterium bifidum* (bb12) to its seed. The presence of bacteria did not affect the garden cress growth rate. The viability of *Bifidobacterium* during the 3 days of storage was more than 10^6 CFU/g and the garden cress retained its probiotic properties. A lower pH and higher acidity in the probiotic sample were reported in comparison with the control sample which is related to the presence of bacteria, and its acid production but no changes in dry matter percentage were observed. Also, no significant increase was found in vitamin C and fat content of garden cress and both samples had almost equal amounts of these compounds. The viability of bacteria in simulated gastrointestinal conditions was assessed, and the results showed the resistance of *Bifidobacterium* to SGI condition which is an important factor for probiotic foods to exert their health-beneficial effects. The comparison of the sensory evaluation of the control and the probiotic garden cress showed the relative equality of the examined parameters and the final product had a high sensory acceptability. The antifungal activity of *Bifidobacterium* was also examined and 15% of fungal growth inhibition against *Rhizoctonia solani* was reported, which indicates the relatively weak effect of *Bifidobacterium* against this plant pathogen.

Author contributions

Golnaz Shambayati: Methodology, Conceptualization, Investigation, Writing – original draft. Resources
Mojtaba Mohammadzadeh Vazifeh: Conceptualization, Writing – review and editing, Validation. Methodology, Resources, Project administration
Seyed Masoud Hosseini: Project administration, Validation.

Hasan Hajjami Barkousaraei: Investigation, Methodology

References

[1] Yerlikaya O. Starter cultures used in probiotic dairy product preparation and popular probiotic dairy drinks. Food Science and Technology. 2014;34:221-9. https://doi.org/10.1590/fst.2014.0050

[2] Lillo-Pérez S, Guerra-Valle M, Orellana-Palma P, Petzold G. Probiotics in fruit and vegetable matrices: Opportunities for nondairy consumers. Lwt. 2021 Nov 1;151:112106. https://doi.org/10.1016/j.lwt.2021.112106

[3] Casarotti SN, Carneiro BM, Penna AL. Evaluation of the effect of supplementing fermented milk with quinoa flour on probiotic activity. Journal of dairy science. 2014 Oct 1;97(10):6027-35. https://doi.org/10.3168/jds.2014-8197

[4] Gupta S, Abu-Ghannam N. Probiotic fermentation of plant based products: possibilities and opportunities. Critical reviews in food science and nutrition. 2012 Feb 1;52(2):183-99. https://doi.org/10.1080/10408398.2010.499779

[5] Küçükgöz K, Trząskowska M. Nondairy probiotic products: Functional foods that require more attention. Nutrients. 2022 Feb 10;14(4):753. https://doi.org/10.3390/nu14040753

[6] Valero-Cases E, Cerdá-Bernad D, Pastor JJ, Frutos MJ. Non-dairy fermented beverages as potential carriers to ensure probiotics, prebiotics, and bioactive compounds arrival to the gut and their health benefits. Nutrients. 2020 Jun 3;12(6):1666. https://doi.org/10.3390/nu12061666

[7] Vasudha S, Mishra HN. Nondairy probiotic beverages. International Food Research Journal. 2013;20(1):7. http://www.ifrj.upm.edu.my/

[8] Pignattelli S, Broccoli A, Renzi M. Physiological responses of garden cress (L. sativum) to different types of microplastics. Science of the Total Environment. 2020 Jul 20;727:138609. https://doi.org/10.1016/j.scitotenv.2020.138609

[9] Mali RG, Mahajan SG, Mehta AA. Lepidium sativum (Garden cress): a review of contemporary literature and medicinal properties. Advances in Traditional Medicine. 2007;7(4):331-5. https://doi.org/10.3742/OPEM.2007.7.4.331

[10] Arepally D, Ravula SR, Goswami TK. Applications of Probiotics in Dairy Food Products. InEngineering Practices for Milk Products 2019 Sep 30 (pp. 351-363). Apple Academic Press.

[11] Cao D, Lutz A, Hill CB, Callahan DL, Roessner U. A quantitative profiling method of phytohormones and other metabolites applied to barley roots subjected to salinity stress. Frontiers in Plant Science. 2017 Jan 10;7:233111. https://doi.org/10.3389/fpls.2016.02070

[12] Ali MR, Parmar A, Niedbała G, Wojciechowski T, Abou El-Yazied A, El-Gawad HG, Nahhas NE, Ibrahim MF, El-Mogy MM. Improved shelf-life and consumer acceptance of fresh-cut and fried potato strips by an edible coating of garden cress seed mucilage. Foods. 2021 Jul 2;10(7):1536. https://doi.org/10.3390/foods10071536

[13] Son SH, Yang SJ, Jeon HL, Yu HS, Lee NK, Park YS, Paik HD. Antioxidant and immunostimulatory effect of potential probiotic Lactobacillus paraplantarum SC61 isolated from Korean traditional fermented food, jangajji. Microbial Pathogenesis. 2018 Dec 1;125:486-92. https://doi.org/10.1016/j.micpath.2018.10.018

[14] Temprano, F., Albareda, M., Camacho, M. *et al.* Survival of several *Rhizobium/Bradyrhizobium* strains on different inoculant formulations and inoculated seeds. *Int Microbiol* 5, 81–86 (2002). https://doi.org/10.1007/s10123-002-0067-y. https://doi.org/10.1007/s10123-002-0067-y

[15] Khalil SE, Yousef RM. Interaction effects of different soil moisture levels, arbuscular mycorrhizal fungi and three phosphate levels on: I-Growth, Yield and Photosynthetic Activity of Garden Cress (Lepidium sativum L.) plant. International Journal of Advanced Research. 2014;2(6):723-37. http://www.journalijar.com/

[16] Maila MP, Cloete TE. Germination of Lepidium sativum as a method to evaluate polycyclic aromatic hydrocarbons (PAHs) removal from contaminated soil. International Biodeterioration & Biodegradation. 2002 Sep 1;50(2):107-13. https://doi.org/10.1016/S0964-8305(02)00059-8

[17] Ciesielska K, Ciesielski W, Kulawik D, Oszczęda Z, Tomasik P. Cultivation of cress involving water treated under different atmospheres with low-temperature, low-pressure glow plasma of low frequency. Water. 2020 Jul 30;12(8):2152. https://doi.org/10.3390/w12082152

[18] Bansal S, Mangal M, Sharma SK, Gupta RK. Non-dairy based probiotics: A healthy treat for intestine. Critical reviews in food science and nutrition. 2016 Aug 17;56(11):1856-67. https://doi.org/10.1080/10408398.2013.790780

[19] OECD guidelines for the testing of chemicals

[20] Erkovan HI, Tan M, Halitligil MB, Kislal H. Performance of white-clover grasses mixtures: Part-I Dry matter production, botanical composition, nitrogen use efficient, nitrogen rate and yield. Asian Journal of Chemistry. 2008 Jul 1;20(5):4071.

[21] Ajdanian L, Babaei M, Aroiee H. The growth and development of cress (Lepidium sativum) affected by blue and red light. Heliyon. 2019 Jul 1;5(7). https://doi.org/10.1016/j.heliyon.2019.e02109

[22] Bao Y, Zhang Y, Zhang Y, Liu Y, Wang S, Dong X, Wang Y, Zhang H. Screening of potential probiotic properties of Lactobacillus fermentum isolated from traditional dairy products. Food control. 2010 May 1;21(5):695-701. https://doi.org/10.1016/j.foodcont.2009.10.010

[23] Di Stefano V, Bongiorno D, Buzzanca C, Indelicato S, Santini A, Lucarini M, Fabbrizio A, Mauro M, Vazzana M, Arizza V, Durazzo A. Fatty acids and triacylglycerols profiles from Sicilian (cold pressed vs. soxhlet) grape seed oils. Sustainability. 2021 Nov 25;13(23):13038. *Sustainability*, *13*(23), 13038. https://doi.org/10.3390/su132313038

[24] ISO 6557/2-1984: Fruits, vegetables and derived products - Determination of ascorbic acid - Part 2: Routine methods.

[25] AdebayoTayo B, Akpeji S. Probiotic viability, physicochemical and sensory properties of probiotic pineapple juice. Fermentation. 2016 Dec 8;2(4):20. https://doi.org/10.3390/fermentation2040020

[26] Pretscher J, Fischkal T, Branscheidt S, Jäger L, Kahl S, Schlander M, Thines E, Claus H. Yeasts from different habitats and their potential as biocontrol agents. Fermentation. 2018 Apr 24;4(2):31. https://doi.org/10.3390/fermentation4020031

[27] Pakbin B, Razavi SH, Mahmoudi R, Gajarbeygi P. Producing probiotic peach juice. Biotechnology and health sciences. 2014. http://dx.doi.org/10.17795/bhs-24683

[28] Pimentel TC, Madrona GS, Garcia S, Prudencio SH. Probiotic viability, physicochemical characteristics and acceptability during refrigerated storage of clarified apple juice supplemented with Lactobacillus paracasei ssp. paracasei and oligofructose in different package type. LWT-Food science and Technology. 2015 Sep 1;63(1):415-22. https://doi.org/10.1016/j.lwt.2015.03.009

[29] Min M, Bunt CR, Mason SL, Bennett GN, Hussain MA. Effect of non-dairy food matrices on the survival of probiotic bacteria during storage. Microorganisms. 2017 Aug 1;5(3):43. https://doi.org/10.3390/microorganisms5030043

[30] Kataria A, Achi SC, Halami PM. Effect of encapsulation on viability of Bifidobacterium longum CFR815j and physiochemical properties of ice cream. Indian journal of microbiology. 2018 Jun;58:248-51. https://doi.org/10.1007/s12088-018-0720-6

[31] Świeca M, Kordowska-Wiater M, Pytka M, Gawlik-Dziki U, Bochnak J, Złotek U, Baraniak B. Lactobacillus plantarum 299V improves the microbiological quality of legume sprouts and effectively survives in these carriers during cold storage and in vitro digestion. PLoS One. 2018 Nov 21;13(11):e0207793.

[32] Oliveira ME, Garcia EF, Queiroga RD, Souza EL. Technological, physicochemical and sensory characteristics of a Brazilian semi-hard goat

cheese (coalho) with added probiotic lactic acid bacteria. Scientia Agricola. 2012;69:370-9. https://doi.org/10.1590/S0103-90162012000600005
[33] Vinderola CG, Mocchiutti P, Reinheimer JA. Interactions among lactic acid starter and probiotic bacteria used for fermented dairy products. Journal of Dairy science. 2002 Apr 1;85(4):721-9. https://doi.org/10.3168/jds.S0022-0302(02)74129-5
[34] Tripathi MK, Giri SK. Probiotic functional foods: Survival of probiotics during processing and storage. Journal of functional foods. 2014 Jul 1;9:225-41. https://doi.org/10.1016/j.jff.2014.04.030
[35] Mohan A, Hadi J, Gutierrez-Maddox N, Li Y, Leung IK, Gao Y, Shu Q, Quek SY. Sensory, microbiological and physicochemical characterisation of functional manuka honey yogurts containing probiotic Lactobacillus reuteri DPC16. Foods. 2020 Jan 19;9(1):106. https://doi.org/10.3390/foods9010106
[36] Casarotti SN, Todorov SD, Penna AL. Effect of different matrices on probiotic resistance to in vitro simulated gastrointestinal conditions. International journal of dairy technology. 2015 Nov;68(4):595-601. https://doi.org/10.1111/1471-0307.12215
[37] Chávarri M, Marañón I, Ares R, Ibáñez FC, Marzo F, del Carmen Villarán M. Microencapsulation of a probiotic and prebiotic in alginate-chitosan capsules improves survival in simulated gastro-intestinal conditions. International journal of food microbiology. 2010 Aug 15;142(1-2):185-9. https://doi.org/10.1016/j.ijfoodmicro.2010.06.022
[38] CHACKOSHIAN KA, Shojaosadati SA. Improvement of probiotic survival in fruit juice and under gastrointestinal conditions using pectin-nanochitin-nanolignocellulose as a novel prebiotic gastrointestinal-resistant matrix. https://doi.org/10.22037/afb.v4i3.17337
[39] Popova T. Effect of probiotics in poultry for improving meat quality. Current Opinion in Food Science. 2017 Apr 1;14:72-7. https://doi.org/10.1016/j.cofs.2017.01.008
[40] Favaro-Trindade CS, Bernardi S, Bodini RB, De Carvalho Balieiro JC, De Almeida E. Sensory acceptability and stability of probiotic microorganisms and vitamin C in fermented acerola (Malpighia emarginata DC.) ice cream. Journal of Food Science. 2006 Aug;71(6):S492-5. https://doi.org/10.1111/j.1750-3841.2006.00100.x
[41] Flores-Félix JD, Silva LR, Rivera LP, Marcos-García M, García-Fraile P, Martínez-Molina E, Mateos PF, Velázquez E, Andrade P, Rivas R. Plants probiotics as a tool to produce highly functional fruits: the case of Phyllobacterium and vitamin C in strawberries. PLoS One. 2015 Apr 15;10(4):e0122281. https://doi.org/10.1371/journal.pone.0122281

[42] Ma X, Wang X, Cheng J, Nie X, Yu X, Zhao Y, Wang W. Microencapsulation of Bacillus subtilis B99-2 and its biocontrol efficiency against Rhizoctonia solani in tomato. Biological control. 2015 Nov 1;90:34-41. https://doi.org/10.1016/j.biocontrol.2015.05.013
[43] Gerbaldo GA, Barberis C, Pascual L, Dalcero A, Barberis L. Antifungal activity of two Lactobacillus strains with potential probiotic properties. FEMS microbiology letters. 2012 Jul 1;332(1):27-33. https://doi.org/10.1111/j.1574-6968.2012.02570.x
[44] Coman MM, Cecchini C, Verdenelli MC, Silvi S, Orpianesi C, Cresci A. Functional foods as carriers for SYNBIO®, a probiotic bacteria combination. International Journal of Food Microbiology. 2012 Jul 16;157(3):346-52.
[45] Afzaal M, Khan AU, Saeed F, Arshad MS, Khan MA, Saeed M, Maan AA, Khan MK, Ismail Z, Ahmed A, Tufail T. Survival and stability of free and encapsulated probiotic bacteria under simulated gastrointestinal conditions and in ice cream. Food Science & Nutrition. 2020 Mar;8(3):1649. https://doi.org/10.1002/fsn3.1451

 Click here to access/download;Figure;figures.docx

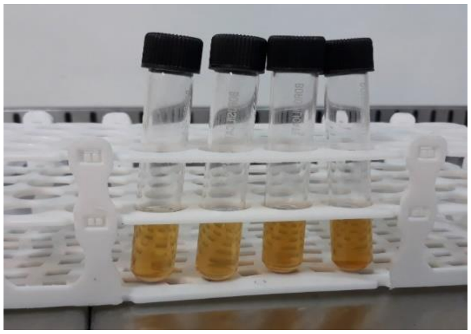

Fig 1. **Inoculum preparation.** The final turbidity of the inoculum was equal to 0.5 McFarland and its volume was 24 ml. This was done by centrifugation of primary culture containing *Bifidobacterium bifidum (*bb12*)* and spectroscopy.

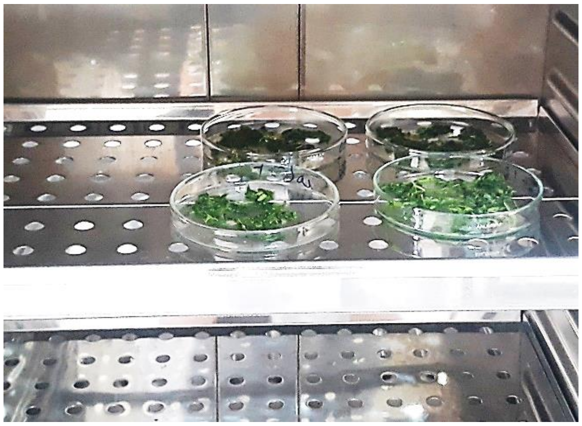

Fig 2. Dry matter measurement

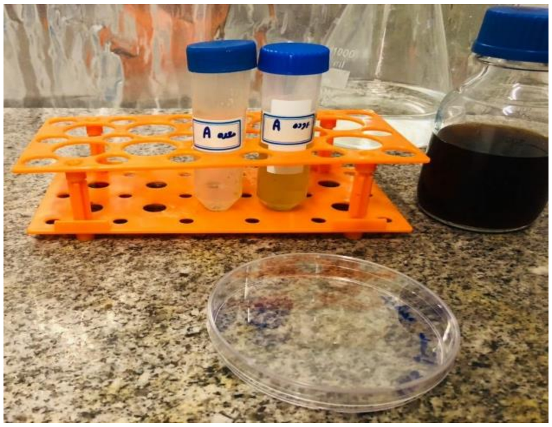

Fig 3. Simulated gastrointestinal conditions preparation. The tubes from left to right, show the gastric and the intestinal conditions. MRS agar medium is used to detect the bacterial growth in these conditions.

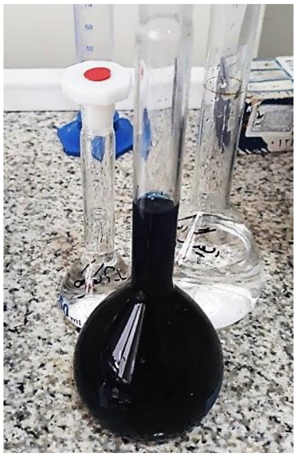

Fig 4. Ascorbic acid solution, 2,6-dichlorophenol-indophenol solution and Oxalic acid are shown respectively from left to right. The Vitamin C content was determined by using these solutions and titration with 2,6-dichlorophenol-indophenol solution.

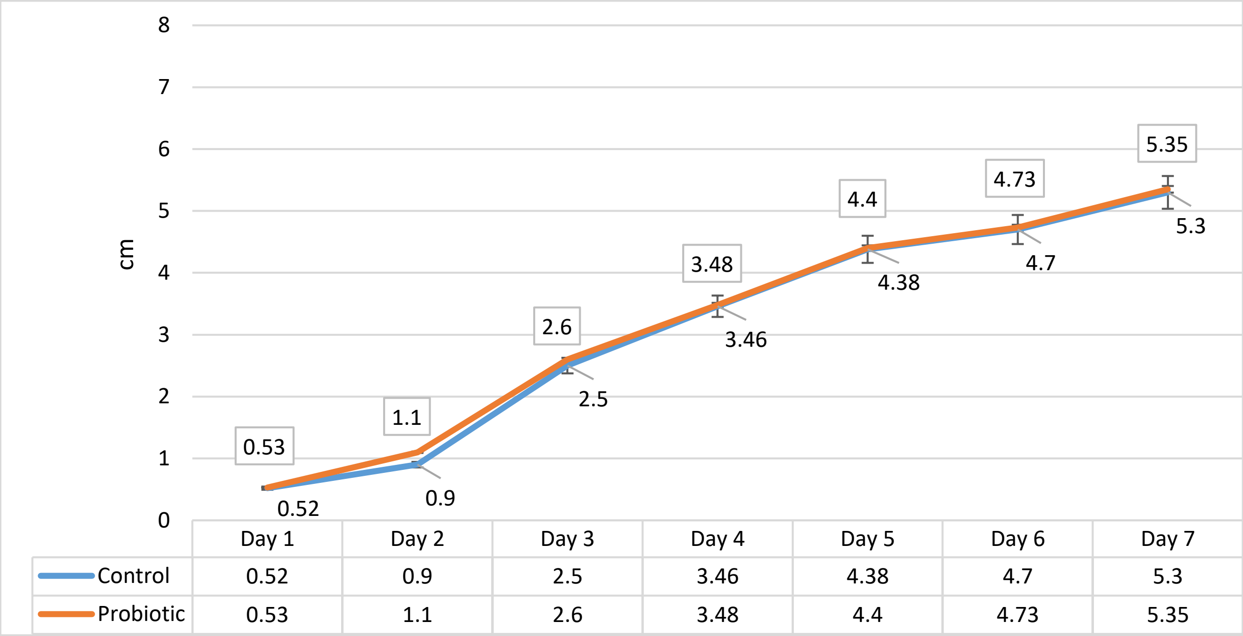

Fig 5. Growth rate comparison of two probiotic and control samples

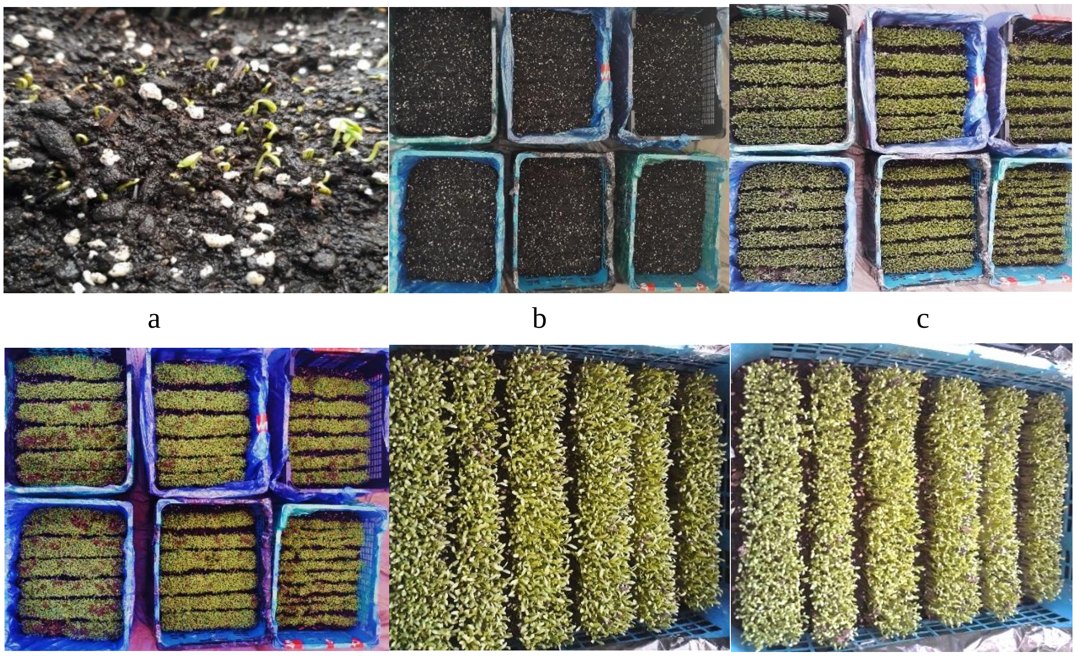

a b c d e f

Fig 6. Plant growth process from day 1 to 6. a: day 1 (24 hours after sowing). Seed germination. The average height of *bifidobacterium* – containing plant and the control one was respectively, 0.53 cm and 0.52 cm. b: day 2. The above vases show the *bifidobacterium* – containig plants and the below ones show the control plants. The average height of probiotic and the control plant on this day was 1.1 cm 0.9 cm respectively. c: day 3. Probiotic plant (2.6 cm), control plant (2.5 cm). d: day 4. Probiotic plant (3.48 cm), control plant (3.46 cm). e: day 5. Probiotc plant (4.4 cm), control plant (4.38 cm). f: day 6. Probiotic plant (4.73 cm), control plant (4.7 cm).

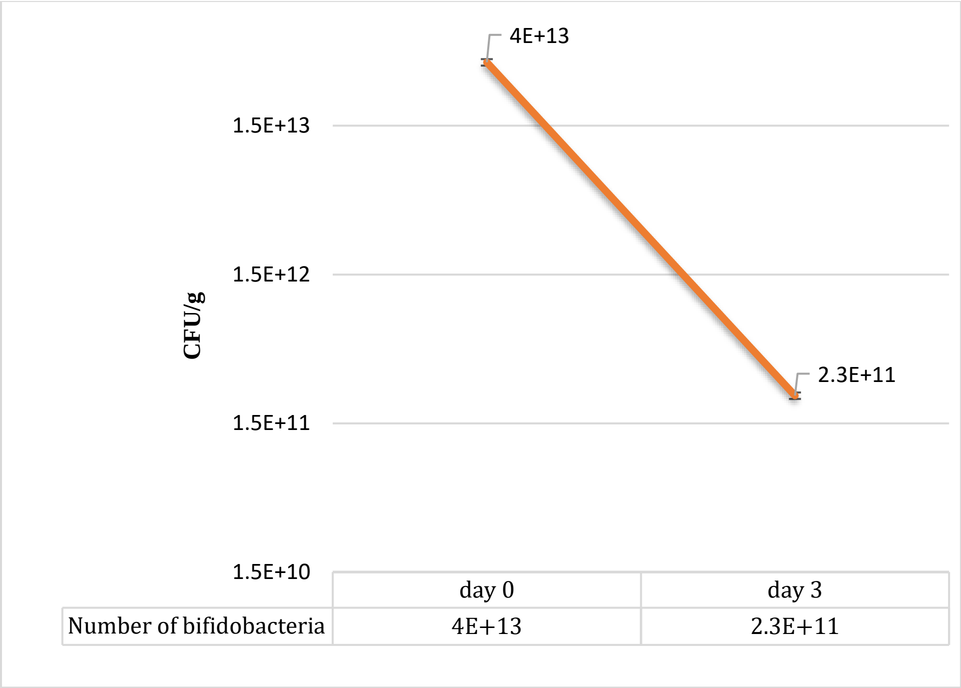

Fig 7. Changes in the number of *Bifidobacteria* from days 0 to 3. A decrease in the bacterial cells number is observed but the product is still a probiotic vegetable

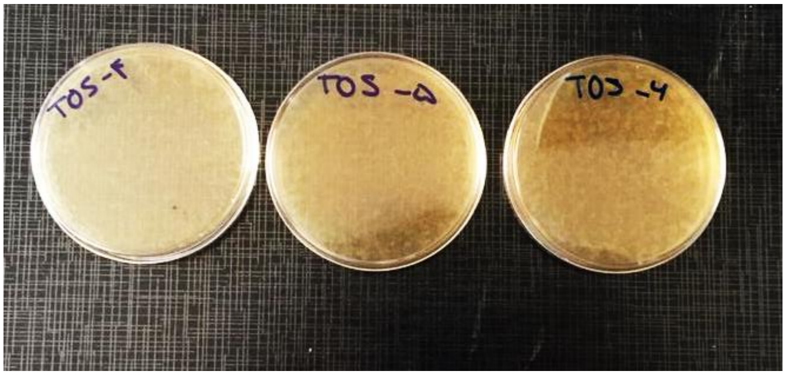

Fig 8. Colony count result

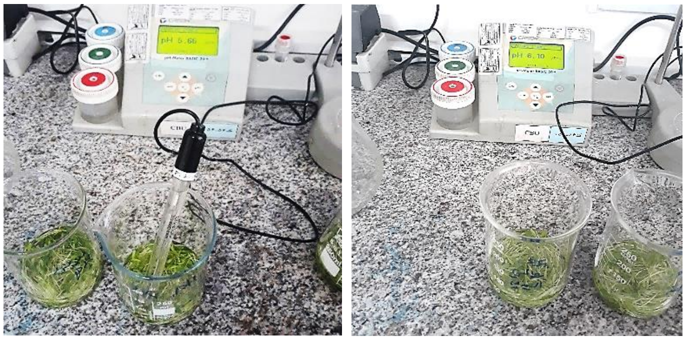

Fig 9. pH measurement of the probiotic and the control sample.

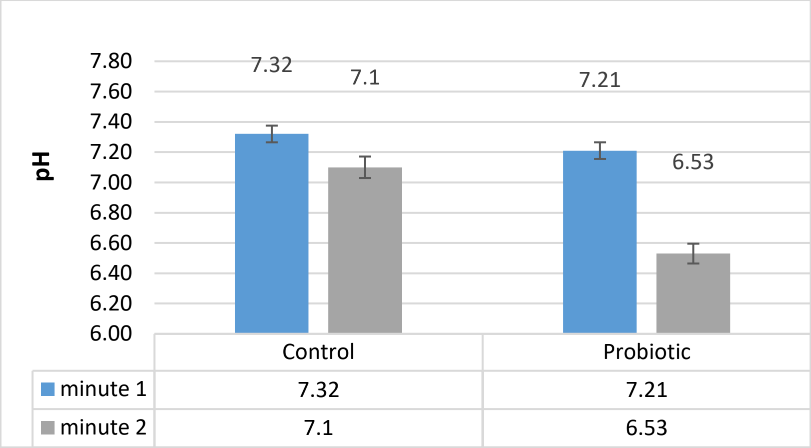

Fig 10. pH values of two samples at minutes 1 and 2

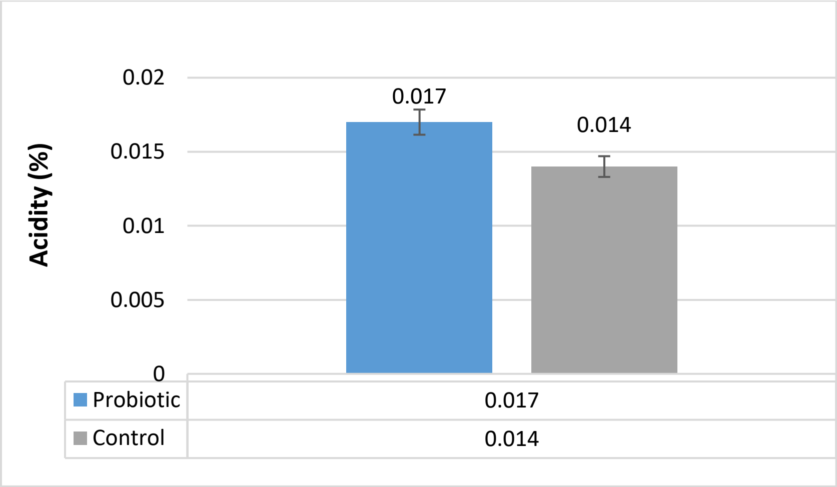

Fig 11. The acidity difference of two samples.

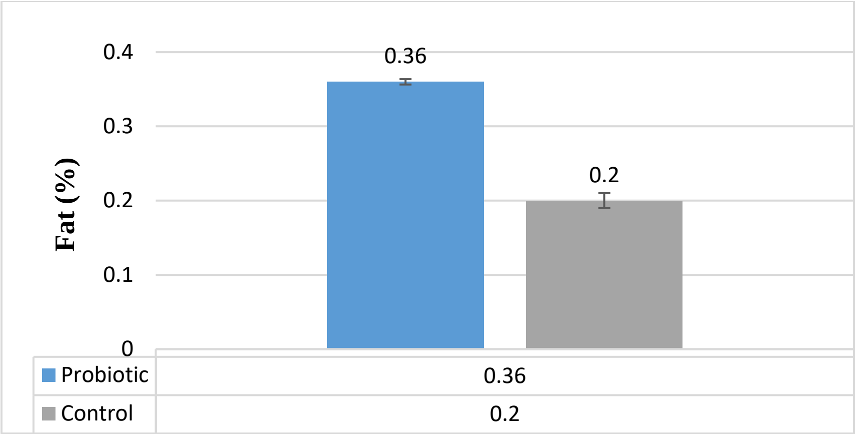

Fig 12. Fat content difference in two samples

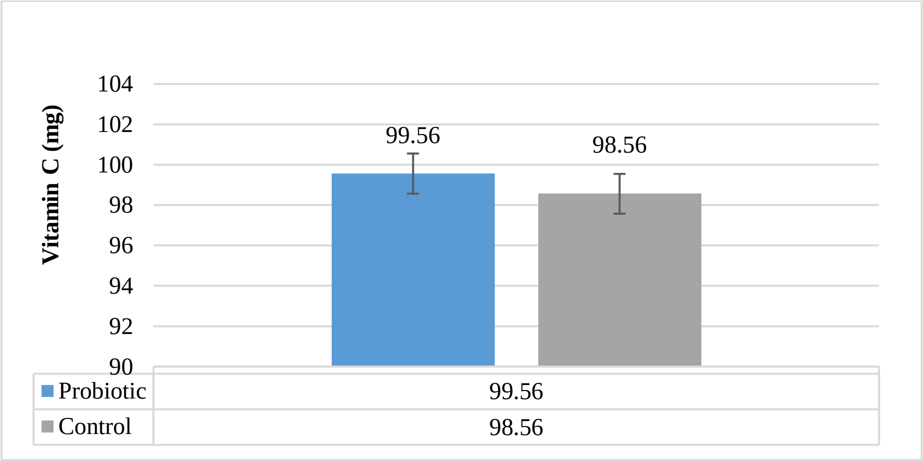

Fig 13. Vitamin C content of the probiotic and the control samples.

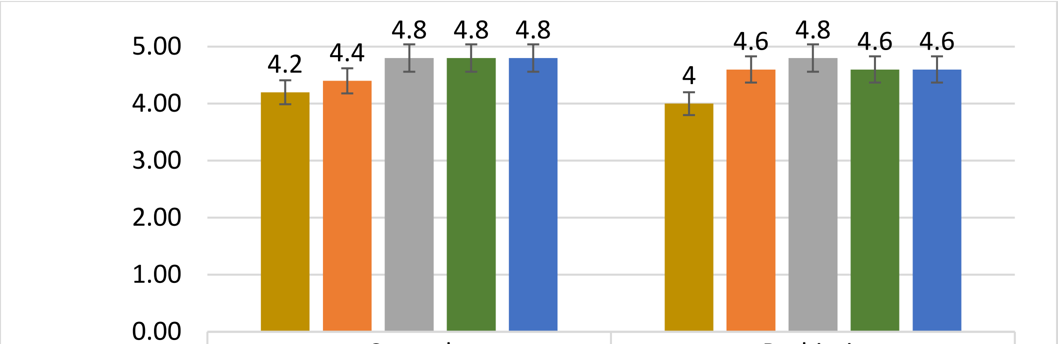

	Control	Probiotic
Aroma	4.2	4
Taste	4.4	4.6
Texture	4.8	4.8
Color	4.8	4.6
Appearance	4.8	4.6

Fig 14. Sensory evaluation results as an average of five determinations.
