# Supplementary material for: Production of probiotic garden cress (Lepidium Sativum) using Bifidobacterium Bifidum and its evaluation of nutritional value, biocontrol and growth rate ability
Source: PLoS One. 2025 Jun 4;20(6):e0322552. doi: 10.1371/journal.pone.0322552 (PMC12136354; doi:10.1371/journal.pone.0322552)
Supplement: Attachment — Submitted filename: plos one reviewer.docx [file pone.0322552.s016.docx]

I have read the manuscript entitled

‘Production of probiotic garden cress (Lepidium Sativum) using Bifidobacterium

Bifidum and its evaluation of nutritional value, bio control and growth rate’ thoroughly. The manuscript is discussing an interesting topic; generation of probiotic garden seeds and assessment of bifidobacteria's viability and resistance in gastrointestinal environments in addition, analyses of its organoleptic qualities, growth rate, and effect on vitamin C content were conducted.

- Where is the key word?
- SGI must be written fully for the first time in abstract.
- Line 93 SGI can be written in abbreviated form as it is mentioned before.
- Line 123 correct the word moistens.
- Line 175 they were determined instead of and were determined.
- Why the authors didn’t make different intervals in determination of total viable count for studying the effect of stomach condition, also why they didn’t make different PH values?
- Why the authors didn’t make different intervals in determination of total viable count for studying the effect of SGI with different concentration for bile condition?
- Line 220 write the name of PDA media (Potato Dextrose Agar) media fully.
- Statistical Analysis is required.
- Conclusion: The whole conclusion should be summarized and include recommendation with mentioning the most important finding not to repeat all the result that presented in detail in the result section.
