# Supplementary material for: Production of probiotic garden cress (Lepidium Sativum) using Bifidobacterium Bifidum and its evaluation of nutritional value, biocontrol and growth rate ability
Source: PLoS One. 2025 Jun 4;20(6):e0322552. doi: 10.1371/journal.pone.0322552 (PMC12136354; doi:10.1371/journal.pone.0322552)
Supplement: Attachment — Submitted filename: Revised PONE-D-24-18993_reviewer.docx [file pone.0322552.s017.docx]

**PLOS ONE**

### Production of probiotic garden cress (Lepidium Sativum) using Bifidobacterium Bifidum and its evaluation of nutritional value, biocontrol and growth rate ability

--Manuscript Draft--

| **Manuscript Number:** | PONE-D-24-18993 |
| --- | --- |
| **Article Type:** | Research Article |
| **Full Title:** | Production of probiotic garden cress (Lepidium Sativum) using Bifidobacterium Bifidum and its evaluation of nutritional value, biocontrol and growth rate ability |
| **Short Title:** | Production of probiotic vegetable |
| **Corresponding Author:** | Golnaz Shambayati  University of Science and Culture  Tehran, Tehran IRAN, ISLAMIC REPUBLIC OF |
| **Keywords:** | Non-dairy probiotic, Garden cress, Bifidobacterium bifidum, bacterial inoculation, probiotic vegetable |
| **Abstract:** | Probiotics are one of the most beneficial elements in human health. Several studies have confirmed the health benefits of probiotics.  This research aimed to produce probiotic garden cress using Bifidobacterium bifidum and to investigate its nutritional value, biocontrol, and growth rate.  Bifidobacterium bifidum (bb12) was inoculated into sterile cress seeds and the probiotic plant culture was done on days 0 and 3 after harvesting, in a TOS medium containing MUP (Mupirisin antibiotic). The chemical properties (such as pH, acidity, and dry matter) of both samples were determined and the comparison of growth rate, fat content, vitamin C, and organoleptic properties was done. Bacterial survival in SGI conditions and its antifungal effect on Rhizoctonia solani were also analyzed.  The number of bacteria on days 0 and 3 was CFU/g and 2.3 CFU/g respectively, which confirms the probiotic nature of the product ( bacteria). However, no significant difference was observed in the physicochemical parameters. Bacterial viability was reported as 6.3 CFU/g in simulated gastric condition and as CFU/g in simulated intestine condition, and the antifungal effect of Bifidobacterium on R. solani was 15%. |
| **Order of Authors:** | Golnaz Shambayati |
|  | Mojtaba Mohammadzadeh Vazifeh |
|  | Seyed Masoud Hosseini |
|  | Hasan Hajjami Barkousaraei |
| **Additional Information:** |  |
| **Question** | **Response** |
| **Financial Disclosure**  Enter a financial disclosure statement that describes the sources of funding for the work included in this submission. Review the [submission guidelines](http://journals.plos.org/plosone/s/submission-guidelines#loc-financial-disclosure-statement) for detailed requirements. View published research articles from [*PLOS ONE*](https://journals.plos.org/plosone/) for specific examples.  This statement is required for submission and **will appear in the published article** if the submission is accepted. Please make sure it is accurate. | The author(s) received no specific funding for this work. |

| **Funded studies**  Enter a statement with the following details:   - Initials of the authors who received each award - Grant numbers awarded to each author - The full name of each funder - URL of each funder website - Did the sponsors or funders play any role in the study design, data collection and analysis, decision to publish, or preparation of the manuscript? |  |
| --- | --- |
| Did you receive funding for this work? |  |
| **Competing Interests**  Use the instructions below to enter a competing interest statement for this submission. On behalf of all authors, disclose any [competing interests](http://journals.plos.org/plosone/s/competing-interests) that could be perceived to bias this work—acknowledging all financial support and any other relevant financial or non- financial competing interests.  This statement is required for submission and **will appear in the published article** if the submission is accepted. Please make sure it is accurate and that any funding sources listed in your Funding Information later in the submission form are also declared in your Financial Disclosure statement.  View published research articles from  [*PLOS ONE*](http://journals.plos.org/plosone/) for specific examples. | The authors have declared that no competing interests exist. |

| **NO authors have competing interests**  Enter: *The authors have declared that no competing interests exist.*  **Authors with competing interests**  Enter competing interest details beginning with this statement:  *I have read the journal's policy and the authors of this manuscript have the following competing interests: [insert competing interests here]* |  |
| --- | --- |
| * typeset |  |
| **Ethics Statement**  Enter an ethics statement for this submission. This statement is required if the study involved:   - Human participants - Human specimens or tissue - Vertebrate animals or cephalopods - Vertebrate embryos or tissues - Field research   Write "N/A" if the submission does not require an ethics statement.  General guidance is provided below. Consult the [submission guidelines](http://journals.plos.org/plosone/s/submission-guidelines#loc-guidelines-for-specific-study-types) for detailed instructions. **Make sure that all information entered here is included in the Methods section of the manuscript.** | N/A |

| **Format for specific study types**  **Human Subject Research (involving human participants and/or tissue)**   - Give the name of the institutional review board or ethics committee that approved the study - Include the approval number and/or a statement indicating approval of this research - Indicate the form of consent obtained (written/oral) or the reason that consent was not obtained (e.g. the data were analyzed anonymously)   **Animal Research (involving vertebrate animals, embryos or tissues)**   - Provide the name of the Institutional Animal Care and Use Committee (IACUC) or other relevant ethics board that reviewed the study protocol, and indicate whether they approved this research or granted a formal waiver of ethical approval - Include an approval number if one was obtained - If the study involved *non-human primates*, add *additional details* about animal welfare and steps taken to ameliorate suffering - If anesthesia, euthanasia, or any kind of animal sacrifice is part of the study, include briefly which substances and/or methods were applied   **Field Research**  Include the following details if this study involves the collection of plant, animal, or other materials from a natural setting:   - Field permit number - Name of the institution or relevant body that granted permission |  |
| --- | --- |
|  |  |
| **Data Availability**  Authors are required to make all data underlying the findings described fully available, without restriction, and from the time of publication. PLOS allows rare exceptions to address legal and ethical concerns. See the [PLOS Data Policy](http://journals.plos.org/plosone/s/data-availability) and [FAQ](http://journals.plos.org/plosone/s/data-availability#loc-faqs-for-data-policy) for detailed information. | Yes - all data are fully available without restriction |

| A Data Availability Statement describing where the data can be found is required at submission. Your answers to this question constitute the Data Availability Statement and **will be published in the article**, if accepted. |  |
| --- | --- |
| **Important:** Stating ‘data available on request from the author’ is not sufficient. If your data are only available upon request, select ‘No’ for the first question and explain your exceptional situation in the text box. |  |
| Do the authors confirm that all data underlying the findings described in their manuscript are fully available without restriction? |  |
| **Describe where the data may be found in full sentences. If you are copying our sample text, replace any instances of XXX with the appropriate details.**   - If the data are **held or will be held in a public repository**, include URLs, accession numbers or DOIs. If this information will only be available after acceptance, indicate this by ticking the box below. For example: *All XXX files are available from the XXX database (accession number(s) XXX, XXX.*). - If the data are all contained **within the manuscript and/or Supporting Information files**, enter the following: *All relevant data are within the manuscript and its Supporting Information files.* - If neither of these applies but you are able to provide **details of access elsewhere**, with or without limitations, please do so. For example:   *Data cannot be shared publicly because of [XXX]. Data are available from the XXX Institutional Data Access / Ethics Committee (contact via XXX) for researchers who meet the criteria for access to confidential data.*  *The data underlying the results presented in the study are available from (include the name of the third party* | The data underlying the results presented in the study are available from (Golnaz Shambayati/gshambayati97@gmail.com). |

| *and contact information or URL).*   - This text is appropriate if the data are owned by a third party and authors do not have permission to share the data.   * typeset |  |
| --- | --- |
| Additional data availability information: | Tick here if the URLs/accession numbers/DOIs will be available only after acceptance of the manuscript for publication so that we can ensure their inclusion before publication. |

Manuscript	[Click here to access/download;Manuscript;Final (1) - Copy.docx](https://www2.cloud.editorialmanager.com/pone/download.aspx?id=36352841&guid=a849a650-e5dc-493a-97ee-e06bf8e580ec&scheme=1)

# 1 Title

2 Production of probiotic garden cress (*Lepidium Sativum*) using *Bifidobacterium*
3 *Bifidum* and its evaluation of nutritional value, biocontrol and growth rate ability
4 Golnaz	Shambayati^1^,	Mojtaba	Mohammadzadeh	Vazifeh^1*^,	Seyed	Masoud
5 Hosseini^2^, Hasan Hajjami Barkousaraei^1^
6 ^1^ Faculty of Modern Biological Sciences and Technologies, University of Science
7 and Culture, Tehran, Iran
8 ^2^ Faculty of Biological Sciences and New Technologies, SBU, Tehran, Iran

### 9 *Corresponding Author:

10 Mojtaba Mohammadzadeh Vazifeh
11 Email: [mohammadzadeh.vazifeh@usc.ac.ir](mailto:mohammadzadeh.vazifeh@usc.ac.ir)

12

13

14

15

16

17

18

19

20

21

22

23

24

25

26

27

28

29

# 30 Abstract

31 Probiotics are one of the most beneficial elements in human health. Several studies
32 have confirmed the health benefits of probiotics.
33 This research aimed to produce probiotic garden cress using *Bifidobacterium*
34 *bifidum* and to investigate its nutritional value, biocontrol, and growth rate.
35 *Bifidobacterium bifidum* (bb12) was inoculated into sterile cress seeds and the
36 probiotic plant culture was done on days 0 and 3 after harvesting, in a TOS medium
37 containing MUP (Mupirisin antibiotic). The chemical properties (such as pH,
38 acidity, and dry matter) of both samples were determined and the comparison of
39 growth rate, fat content, vitamin C, and organoleptic properties was done. Bacterial
40 survival in SGI conditions and its antifungal effect on *Rhizoctonia solani* were also
41 analyzed.
42 The number of bacteria on days 0 and 3 was 4×10^13^ CFU/g and 2.3×10^11^ CFU/g
43 respectively, which confirms the probiotic nature of the product (>10^6^ bacteria).
44 However, no significant difference was observed in the physicochemical parameters.
45 Bacterial viability was reported as 6.3×10^10^ CFU/g in simulated gastric condition
46 and as 3×10^6^ CFU/g in simulated intestine condition, and the antifungal effect of
47 *Bifidobacterium* on *R. solani* was 15%.

### 48 Abbreviation:

49 SGI: simulated gastrointestinal

50

51

52

53

54

55

56

57

# 58 Introduction

59 The gut microbiota is mostly shaped by the environment, in particular the diet.
60 Probiotics are defined as living microorganisms that affect the health of the host by
61 improving the composition of gut microflora [1]. Consumption of probiotics has
62 beneficial effects on the prevention of various diseases [2]. It is commonly accepted
63 that probiotics must be present at a minimum level, ranging from 10^6^-10^9^ CFU/ml
64 of the product, to be beneficial [3].
65 Common	microorganisms	used	in	probiotic	production	are	predominantly
66 *Lactobacillus*, and *Bifidobacterium* species which produce organic acid and lower
67 the intestinal pH [4]. Probiotic strains should be resistant to gastrointestinal
68 conditions, and be safe for human use [5].
69 Most probiotic foods are milk-based but certain sectors of the population such as
70 those allergic to milk proteins, those who are lactose intolerant, and those who are
71 vegetarian, cannot consume dairy products. Therefore, a need has arisen among
72 consumers for an alternative to fermented dairy products by exploring new non-dairy
73 matrices as probiotics carriers [6], [7]. Fruit and vegetables are nondairy origin
74 matrices and have great potential to preserve probiotic viability either due to their
75 morphology or because they act as substrates or prebiotics [2].
76 *Lepidium sativum*, also known as garden cress, is a fast-growing annual herbaceous
77 plant belonging to the *Brassicaceae* family [8]. It is used in the treatment of cough
78 and bleeding piles. Leaves are mildly stimulant, diuretic, and useful in scorbutic
79 diseases and liver complaints [9].
80 In this research, we tried to produce a probiotic garden cress by inoculating its seeds
81 with *Bifidobacterium bifidum* (bb12) bacteria.

# 82 Materials and methods

## 83 Materials

84 Probiotic cells, *Bifidobacterium bifidium* (bb12), were provided by (Chr-Hansen
85 A/S., Hørsholm, Denmark) and *Rhizoctonia. solani* fungi (IBRC-M 30523) was
86 provided to determine the antifungal activity of *Bifidobacterium*. MRS Broth and
87 MRS Agar mediums (Ibresco Co., Iran) and TOS Agar (Biomark, India) were used
88 for culture activation and *Bifidobacterium* growth. PDA Agar (Merck, Kenilworth,
89 NJ, USA) was used as the main medium for *Rhizoctonia. solani* culture. Hydrogen
90 chloride (HCl), Sodium hydroxide (NaOH), and n-hexane solutions were purchased
91 from (Dr. Mojallali Industrial Chemical Co., Tehran, Iran). Trypsin (Sigma Aldrich
92 Co., St. Louis, MO, USA), Ox gall (Sigma Aldrich) and Pepsin (Sigma Aldrich)
93 were utilized to prepare simulated gastrointestinal conditions.

## 94 Production of probiotic garden cress

### 95 Culture revival

96 The MRS broth medium was sterilized in an autoclave at 121℃ for 15 minutes.
97 Bacteria were cultured in a sterile MRS broth medium and incubated anaerobically

98	at 37℃ for 48-72 hours [10].

### 99 Garden cress seed preparation

100 **Surface sterilization**
101 100 grams of fresh garden cress seeds were purchased from the grocery store and
102 divided equally into two sterile beakers. The seeds were surface sterilized in 70%
103 ethanol for 1 minute, and hypochlorite solution for 10 minutes. Finally, the seeds
104 were rinsed 5 times with sterile distilled water [11].
105 105

### 106 Seed mucilage generation

107 Each seed sample (50 g) was soaked separately in two containers (each containing
108 0.5 liters of sterile distilled water) for 12 hours [12].
109 109

### 110 Seed inoculation

111 The medium suspension containing *Bifidobacterium* strains was separated by
112 centrifugation (Hettich Instruments, Tuttlingen, Germany) at 4500 rpm for 10
113 minutes at room temperature and after being washed twice with PBS buffer (Merck),
114 the bacterial sediment was separated [13]. 50 g of seeds were mixed with the
115 inoculant containing *Bifidobacterium bifidum* at a ratio of 1:100 (inoculant: seed
116 weight). The total volume of the inoculant was 24 ml and its turbidity was equal to
117 0.5 McFarland (1.5×10^8^ CFU/ml) (Fig 1). The seeds were kept in the inoculum for

118	2 hours [14].

119

## 120 Seed planting

121 6 vases (3 vases for each treatment) and cocopeat soil were prepared for planting
122 seeds. 10 kg of soil was added to each vase [15]. 2 liters of water were added to each
123 vase to mois5ten the soil [16]. The plants were watered with 0.5 liters of water twice

124	a day [17].

125

## 126 Plant harvesting

127 Both treatments were harvested one week after planting when the plant height
128 reached 5 cm. Both samples were separately transferred to the laboratory in sterile
129 plastics [18].
130 130

## 131 Comparison of duration and rate of growth

132 This was done by preparing a table and checking the height of the plant daily with a
133 caliper.
134 134

## 135 Bacterial enumeration

136 Bacterial enumeration was done according to the method described by Arepally and
137 Goswami [10] with slight modifications. Five grams of the bacteria-containing
138 sample were washed, cut, and dissolved in a 45 ml PBS solution and the serial
139 dilution was performed. 1 ml of each dilution was cultured in TOS medium
140 containing MUP (Merk) supplement by pour plate method and incubated in an
141 anaerobic jar (Afshar Co., Iran) at 37℃ for 72 hours. The viability of bacteria was
142 examined on days 0 and 3. Samples were kept from day 0 to 3 at refrigerator
143 temperature (4℃) in plastic bags.

## 144 Physicochemical measurements

### 145 pH

146 The pH of both samples was measured according to OECD guidelines for the testing
147 of chemicals. After calibrating the pH meter (Crison Co., France), 5 grams of sample
148 was mixed with 50 milliliters of distilled water, and the pH value was read at minutes

149	1 and 2 [19].

### 150 Acidity

151 The acidity of both samples was measured according to OECD guidelines for the
152 testing of chemicals. To determine the acidity, 5 grams of each sample was titrated
153 with 0.1N sodium hydroxide. The acidity of both samples was measured using the
154 following formula:
155 Acidity (calculated as linoleic acid) = 1.31 t×c_1_/w (%)
156 Where;
157 c_1_= (NaOH). mol/L (normality) of the solution
158 t = volume (mL) of NaOH solution
159 w = weight (g)of a sample [19] 160

## 161 Dry matter percentage measurement

162 Dry matter was determined by drying the weighed samples in an oven (Shimaz, Iran)
163 at 78℃ for 24h (Fig 2). the following formula was used to measure the percentage
164 of dry matter [20], [21]:
165 Percentage of dry matter = fresh sample weight-dry sample weight/fresh sample weight ×100

## 166 Viability of bacteria under simulated gastrointestinal conditions

167 Simulated gastric juice was prepared by adding 0.35 g of pepsin in 100 ml of 0.2%
168 saline and adjusting the pH to 2.0 and 2.5 with hydrochloric acid. Simulated
169 intestinal juice was prepared by adding 0.1 g of trypsin and 1.8 g of bile salts in a
170 sterile solution of 1.1 g of sodium bicarbonate and 0.2 g of sodium chloride in 100
171 ml distilled water and adjusting pH to 8.0 with 0.5 M sodium hydroxide (Fig 3).
172 Both solutions were sterilized with filtering through 0.45 μm filter [22].

## 173 Determination of total viable count

174 The changes in the total number of living bacteria during the treatment with gastric
175 juice (3 hours) and intestinal juice (3 hours) and were determined by the pour plate
176 method using MRS agar on day 3. The plates were incubated anaerobically at 37℃
177 for 48 hours. The number of *Bifidobacterium bifidum* in samples before and after the
178 treatment was counted and compared. The viability of the bacteria was calculated
179 according to the following equation:
180 Survival rate (%) = log CFU 𝑁_1_⁄log CFU 𝑁_0_ ×100
181 Where 𝑁_1_ represents the total viable count of *Bifidobacterium bifidum* after
182 treatment by simulated gastrointestinal juices, and 𝑁_0_ represents the total viable
183 count of *Bifidobacterium bifidum* before treatment [22].
184 184

## 185 Fat content

186 Fat content determination was done according to the method described by Stefano
187 et al. [23] with slight modifications. Three grams of each sample were extracted in
188 n-hexane solvent using a Soxhlet extractor for 6 hours. The extract was filtered and
189 concentrated in a rotary evaporator at 40℃. The amount of extracted oil was
190 obtained using the following formula:
191 Fat percentage = fat weight/sample weight ×100
192 192

## 193 Vitamin C determination

194 The amount of vitamin C was determined according to ISO 6557/2-1984: Fruits,
195 vegetables and derived products - Determination of ascorbic acid - Part 2: Routine
196 methods [24]. The amount of vitamin C was measured by extracting ascorbic acid
197 from the samples, using the oxalic acid (Sigma Aldrich) solution with the acetic acid,
198 and titration with 2,6-dichlorophenol-indophenol (Merk) until a light pink color
199 appeared (Fig 4). Vitamin C content (mg) was calculated according to the following
200 equation:

201	Vitamin C = (V_0_-V_1_)×m_1_⁄m_0_ ×100

202 Where V_0_ represents the volume of dye solution (ml) used for titration, V_1_ represents
203 the volume of the dye solution (ml) used in the control test, m_1_ represents the mass
204 of ascorbic acid (mg) and m_0_ represents the mass of the sample (g) used in the
205 titration test.

## 206 Sensory evaluation

207 The characteristics of Garden cress in terms of taste (spiciness), aroma, texture,
208 color, and appearance were examined by five panelists. The organoleptic evaluation
209 in this research was based on the hedonic sensory evaluation, which is a 5-point
210 method (from most unfavorable to most favorable). Testing was carried out by
211 giving a questionnaire to the panelists with parameters, i.e., color, aroma, taste,
212 texture, and appearance. To achieve quantitative results, the qualitative results
213 obtained from the test were converted into quantitative (parametric) data and results
214 by giving the points from 1 to 5 to properties [25].
215 **Antifungal activity of *Bifidobacterium bifidum***
216 The agar well diffusion method was used to analyze the antifungal activity of
217 bacteria. To prepare the supernatant, *Bifidobacterium bifidum* bacteria were cultured
218 on an MRS broth medium for 48 hours. Then it was centrifuged at 3000 rpm for 15
219 minutes. 1 cc of the supernatant was filtered by a 0.45 μm syringe filter. On the other
220 hand, a plate containing sterile PDA agar medium was prepared and 4 wells with a
221 diameter of 0.6 mm were bored using a sterile punch. 100 μl of the supernatant was
222 added to the wells (until completely filled) and one well was considered as a control
223 sample containing sterile distilled water. An agar plug (0.5×0.5 cm) was removed
224 from the fresh culture of *Rhizoctonia solani*, and placed in the center of the plate and
225 between the wells. The plate was incubated for 4 days at 25℃. The radius of the
226 growth zone (cm) was measured in both samples, and the results were recorded after
227 4 days. Antifungal activity was calculated according to the following formula:

228	FI(%) = R_c_-R_t_⁄R_c_ ×100

229 Where Rc represents the radius of the growth zone in the control and Rt represents
230 the radius of the growth zone in *Bifidobacterium*-containing treatment [26].

# 231 Results

## 232 Comparison of duration and rate of growth

233 The result of this measurement and comparison of the data showed that the addition
234 of *Bifidobacterium* to the plant did not change the rate and time of its growth (Fig
235 5), and both treatments had equal growth under the same planting conditions in terms
236 of light and watering (Fig 6).

## 237 Probiotic count

238 The number of *bifidobacteria* in the garden cress was determined as the average of
239 three counts (Fig 7 and 8). According to recommendations, there should be a
240 minimum of 10^6^ CFU/ml or g of live probiotic bacteria at the time of consumption.
241 Counting *bifidobacteria* in inoculated garden cress shows that plant is indeed a
242 probiotic vegetable.

## 243 Physicochemical properties analysis

244 The pH value of the control sample and the *bifidobacteria* – containing one was
245 determined at minutes one and two (Fig 9). The difference in the pH of the two
246 treatments and the low pH of the probiotic sample can confirm the presence of
247 *Bifidobacterium* (which produces acid) in the garden cress (Fig 10).
248 248
249 To determine the acidity, 5 grams of each sample was measured, and individually
250 titrated with 0.1N sodium hydroxide. The volume of sodium hydroxide used for
251 control and probiotic samples was 0.55 ml and 0.66 ml, respectively (Fig 11). The
252 acidity of each sample was calculated as follows:
253 253

Control= 1.31×0.1×0.55⁄5

= 0.014٪

254 254
255 255
256 256

Probiotic= 1.31×0.1×0.66⁄5

## Dry matter

= 0.017٪

257 The weight of the samples before and after being placed in the oven was measured
258 and the percentage of dry matter was calculated according to the below formula:

259	Control sample = 3.330-0.198⁄3.330 ×100 = 94٪

260	Probiotic sample = 3.320-0.191⁄3.320 ×100 = 94٪

261

262 **The viability of *bifidobacteria* under gastrointestinal conditions**
263 The	number	of	viable	*bifidobacteria*	in	the	garden	cress	reached
264 6.3×10^11^ CFU/g after exposure to the simulated gastric condition. The number of
265 viable *bifidobacteria* was 3×10^6^ CFU/g under simulated intestinal conditions. So,
266 we conclude that the resulting product could survive in the simulated gastrointestinal
267 conditions, which is a significant point because one of the points in the production
268 of probiotic foods is their survival in the human digestive system and resistance to
269 gastric and intestinal conditions.
270 270

## 271 Fat content

272 The amount of fat (%) was determined by measuring the mass of the samples and
273 the Soxhlet extraction apparatus before and after being placed in a Soxhlet extractor
274 and rotary evaporator.
275 The results of this measurement show that the presence of *Bifidobacterium* did not
276 change the amount of fat in garden cress and the percentage of fat in both control
277 and probiotic samples was almost the same (Fig 12).

## 278 Vitamin C content

279 The volume of dye solution used for titration was 0.2 ml, the volume of the solution
280 used in the control test was 0.1 ml, the mass of the ascorbic acid was 50 mg, and the
281 mass of the samples used for the titration was approximately 5 grams (Fig 13). The
282 results show no significant difference and both samples have equal amount of
283 Vitamin C.

## 284 Organoleptic properties analysis

285 Five panelists were done the organoleptic evaluation of both control and probiotic
286 samples, the results of which are shown as the average of each parameter (from 1 to
287 5) in the graph below (Fig 14):
288 **Antifungal activity of *Bifidobacterium bifidum* (bb12)**
289 The radius of the growth zone in the control sample was reported as 3.8 cm, and in
290 the sample containing *Bifidobacterium*, it was reported as 3.2 cm. The percentage of
291 fungi growth inhibition by *Bifidobacterium* was calculated according to the
292 following formula:

293	FI = 3.8-3.2⁄3.8 ×100 = 15٪

# 294 Discussion

295 In confirmation of the obtained results in the enumeration and survival of bacteria
296 and the decrease in the number of them during the storage period, Babak Pakbin et
297 al. [27] observed that the number of *Lactobacillus delbruecki* bacteria in their
298 product reached approximately 10×10^9^ CFU/ml and it was suitable for consumption
299 as a probiotic drink. A decrease in the number of bacteria was observed from weeks
300 0 to 4, but there was no change in the probiotic properties of the product. Tatiana
301 Colombo Pimentel et al. [28] investigated the probiotic viability of apple juice
302 supplemented with two probiotic strains and reported a rapid loss in the number of
303 bacteria during the storage of juices. It has been reported by Min et al. [29] that the
304 food matrix used to inoculate probiotic strains can affect their viability and
305 announced that the population of the three strains used, reduced with increasing
306 storage time. Ankita Kataria et al. [30] reported that it is possible to deliver
307 *Bifidobacterium longum* through ice cream. The legume sprouts and *Lb. plantarum*
308 299V can be successfully combined to form a new probiotic-rich food [31]. The
309 enumeration of the bacteria in the present study showed the essential count for
310 probiotic bacteria to exhibit beneficial effects.
311 We observed a lower pH and higher acidity in the probiotic sample in comparison
312 with the control one, which confirms the presence of *Bifidobacterium* and its acid
313 production but no changes in dry matter were reported. Oliveira et al. [32] reported
314 an increase in dry matter of goat cheese and stated that this could be related to an
315 increase in the percentage of protein. So, we can conclude that changes in dry matter
316 might be observed in dairy probiotic products. Vinderola et al. [33] reported that the
317 pH values in two types of yogurt containing *Bifidobacterium* and *Lactobacillus* were
318 negligible during the refrigerated storage. The garden cress pH in the present study
319 was in the range of 6.5-7.2 which includes the optimum pH for the growth of
320 *Bifidobacterium*. Many research has confirmed that the presence of probiotic strains
321 in products does not have a significant effect on the titratable acidity or may increase
322 it [34]. The decrease in pH value in honey yogurt containing *Lactobacillus reuteri*
323 DPC16 during the 3 weeks of storage was also reported by Anand Mohan et al. [35].
324 The survival of probiotic strains is an important factor for exertion their health
325 benefits. In present study, the viability of *Bifidobacterium* decreased after exposure
326 to SGI condition but still was higher than the minimal range accepted to be beneficial
327 (≥10^6^ CFU/ml or g). This result is similar to those reported by Casarotti et al. [36]
328 who demonstrated that the viability of probiotic strains significantly decreased after
329 exposure to in vitro gastrointestinal conditions. Moreover, Chávarri et al. [37]
330 observed a rapid loss in the number of free bacterial cells in SGI conditions. In a
331 more recent study by Khorasani and Shojaosadati [38] it has been found that the
332 number of viable probiotic bacteria decreased during exposure to SGI conditions.
333 It has been demonstrated by many studies that the fat content of foods provides
334 protection for probiotic bacteria. In the present study, the fat content of probiotic
335 sample was not significantly higher than the control one. Ciesielska et al. [17] have
336 shown that the kind of water is what affects the fat content in garden cress. Popova
337 [39] reported that the addition of probiotic strains reduces the saturated fatty acids
338 and increases the polyunsaturated fatty acids of meat, and thus has a positive effect
339 on it.
340 We also observed that the Vitamin C content was not affected by *Bifidobacterium*
341 and its value difference in both samples was 1 mg (higher in probiotic sample).
342 AdebayoTayo et al. [25] found that the vitamin C content in probiotic pineapple
343 juice had no significant differences during storage but no significant change in
344 Vitamin C content of the fermented Acerolla ice cream was observed by Favaro
345 Tritade et al. [40]. Flores-Félix et al. [41] reported that the vitamin C highly
346 increased in the strawberries inoculated with plant probiotics.
347 The fungal growth inhibition of *Bifidobacterium* against *R. solani* that we observed
348 in present study was 15%. Xin Ma et al. [42] also worked on another probiotic strain,
349 *Bacillus subtilis*, and reported that *B. subtilis* has been used widely in agriculture as
350 a biocontrol agent and has a high antifungal activity against *R. solani*. Gerbaldo et
351 al. [43] have also indicated the antifungal effect of two *Lactobacillus* strains against
352 aflatoxigenic fungal isolate.
353 The organoleptic evaluation showed no difference in *Bifidobacterium –* containing
354 garden cress. Coman et al. [44] worked on four probiotic foods, and reported that no
355 significant differences were observed between probiotic-enriched foods and control
356 foods. Afzaal et al. [45] demonstrated that the taste and the flavor of probiotic and
357 control ice cream were the same and no difference was observed.

# 358 Conclusion

359 In this study, we produced probiotic garden cress by inoculating *Bifidobacterium*
360 *bifidum* (bb12) to its seed. The presence of bacteria did not affect the garden cress
361 growth rate. The viability of *Bifidobacterium* during the 3 days of storage was more
362 than 10^6^ CFU/g and the garden cress retained its probiotic properties. A lower pH
363 and higher acidity in the probiotic sample were reported in comparison with the
364 control sample which is related to the presence of bacteria, and its acid production
365 but no changes in dry matter percentage were observed. Also, no significant increase
366 was found in vitamin C and fat content of garden cress and both samples had almost
367 equal amounts of these compounds. The viability of bacteria in simulated
368 gastrointestinal conditions was assessed, and the results showed the resistance of
369 *Bifidobacterium* to SGI condition which is an important factor for probiotic foods to
370 exert their health-beneficial effects. The comparison of the sensory evaluation of the
371 control and the probiotic garden cress showed the relative equality of the examined
372 parameters and the final product had a high sensory acceptability. The antifungal
373 activity of *Bifidobacterium* was also examined and 15% of fungal growth inhibition
374 against *Rhizoctonia solani* was reported, which indicates the relatively weak effect
375 of *Bifidobacterium* against this plant pathogen.

# 376 Author contributions

377 Golnaz Shambayati: Methodology, Conceptualization, Investigation, Writing –
378 original draft. Resources
379 Mojtaba Mohammadzadeh Vazifeh: Conceptualization, Writing – review and
380 editing, Validation. Methodology, Resources, Project administration
381 Seyed Masoud Hosseini: Project administration, Validation.

| 382 | Hasan Hajjami Barkousaraei: Investigation, Methodology |
| --- | --- |
| 383 |  |
| 384 | **References** |
| 385 | [1] Yerlikaya O. Starter cultures used in probiotic dairy product preparation and |
| 386 | popular probiotic dairy drinks. Food Science and Technology. 2014;34:221-9. |
| 387 | <https://doi.org/10.1590/fst.2014.0050> |
| 388 | [2] Lillo-Pérez S, Guerra-Valle M, Orellana-Palma P, Petzold G. Probiotics in fruit |
| 389 | and vegetable matrices: Opportunities for nondairy consumers. Lwt. 2021 Nov |
| 390 | 1;151:112106. <https://doi.org/10.1016/j.lwt.2021.112106> |
| 391 | [3] Casarotti SN, Carneiro BM, Penna AL. Evaluation of the effect of supplementing |
| 392 | fermented milk with quinoa flour on probiotic activity. Journal of dairy science. |
| 393 | 2014 Oct 1;97(10):6027-35. <https://doi.org/10.3168/jds.2014-8197> |
| 394 | [4] Gupta S, Abu-Ghannam N. Probiotic fermentation of plant based products: |
| 395 | possibilities and opportunities. Critical reviews in food science and nutrition. |
| 396 | 2012 Feb 1;52(2):183-99. <https://doi.org/10.1080/10408398.2010.499779> |
| 397 | [5] Küçükgöz K, Trząskowska M. Nondairy probiotic products: Functional foods |

398	that	require	more	attention.	Nutrients.	2022	Feb	10;14(4):753.

399	<https://doi.org/10.3390/nu14040753>

400 [6] Valero-Cases E, Cerdá-Bernad D, Pastor JJ, Frutos MJ. Non-dairy fermented
401 beverages as potential carriers to ensure probiotics, prebiotics, and bioactive
402 compounds arrival to the gut and their health benefits. Nutrients. 2020 Jun

403	3;12(6):1666. <https://doi.org/10.3390/nu12061666>

404	[7] Vasudha S, Mishra HN. Nondairy probiotic beverages. International Food

405	Research Journal. 2013;20(1):7. <http://www.ifrj.upm.edu.my/>

406	[8] Pignattelli S, Broccoli A, Renzi M. Physiological responses of garden cress (L.

407	sativum) to different types of microplastics. Science of the Total Environment.

408	2020 Jul 20;727:138609. <https://doi.org/10.1016/j.scitotenv.2020.138609>

409	[9] Mali RG, Mahajan SG, Mehta AA. Lepidium sativum (Garden cress): a review

410	of contemporary literature and medicinal properties. Advances in Traditional

411	Medicine. 2007;7(4):331-5. <https://doi.org/10.3742/OPEM.2007.7.4.331>

412 [10]	Arepally D, Ravula SR, Goswami TK. Applications of Probiotics in Dairy
413 Food Products. InEngineering Practices for Milk Products 2019 Sep 30 (pp. 351-
414 363). Apple Academic Press.
415 [11] Cao D, Lutz A, Hill CB, Callahan DL, Roessner U. A quantitative profiling
416 method of phytohormones and other metabolites applied to barley roots
417 subjected to salinity stress. Frontiers in Plant Science. 2017 Jan 10;7:233111.

418	<https://doi.org/10.3389/fpls.2016.02070>

419	[12] Ali MR, Parmar A, Niedbała G, Wojciechowski T, Abou El-Yazied A, El-

420	Gawad HG, Nahhas NE, Ibrahim MF, El-Mogy MM. Improved shelf-life and

421	consumer acceptance of fresh-cut and fried potato strips by an edible coating of

422	garden	cress	seed	mucilage.	Foods.	2021	Jul	2;10(7):1536.

423	<https://doi.org/10.3390/foods10071536>

424	[13] Son SH, Yang SJ, Jeon HL, Yu HS, Lee NK, Park YS, Paik HD. Antioxidant

425	and	immunostimulatory	effect	of	potential	probiotic	Lactobacillus

426	paraplantarum SC61 isolated from Korean traditional fermented food, jangajji.

427	Microbial	Pathogenesis.	2018	Dec	1;125:486-92.

428	<https://doi.org/10.1016/j.micpath.2018.10.018>

429	[14] Temprano, F., Albareda, M., Camacho, M. *et	al.* Survival	of

430	several *Rhizobium/Bradyrhizobium* strains on different inoculant formulations

431	and	inoculated	seeds. *Int	Microbiol* 5, 81–86	(2002). 432	https://doi.org/10.1007/s10123-002-0067-y. [https://doi.org/10.1007/s10123-](https://doi.org/10.1007/s10123-002-0067-y) 433	[002-0067-y](https://doi.org/10.1007/s10123-002-0067-y)

434 [15] Khalil SE, Yousef RM. Interaction effects of different soil moisture levels,
435 arbuscular mycorrhizal fungi and three phosphate levels on: I-Growth, Yield and
436 Photosynthetic	Activity of	Garden	Cress	(Lepidium sativum L.)	plant.
437 International	Journal	of	Advanced	Research.	2014;2(6):723-37.
438 <http://www.journalijar.com/>
439 [16] Maila MP, Cloete TE. Germination of Lepidium sativum as a method to evaluate
440 polycyclic aromatic hydrocarbons (PAHs) removal from contaminated soil.
441 International Biodeterioration & Biodegradation. 2002 Sep 1;50(2):107-13.

442	<https://doi.org/10.1016/S0964-8305(02)00059-8>

443	[17]	Ciesielska K, Ciesielski W, Kulawik D, Oszczęda Z, Tomasik P. Cultivation

444	of cress involving water treated under different atmospheres with low-

445	temperature, low-pressure glow plasma of low frequency. Water. 2020 Jul

446	30;12(8):2152. <https://doi.org/10.3390/w12082152>

447	[18]	Bansal S, Mangal M, Sharma SK, Gupta RK. Non-dairy based probiotics: A

448	healthy treat for intestine. Critical reviews in food science and nutrition. 2016

449	Aug 17;56(11):1856-67. https://doi.org/10.1080/10408398.2013.790780

450	[19] OECD guidelines for the testing of chemicals

451	[20]	Erkovan HI, Tan M, Halitligil MB, Kislal H. Performance of white-clover

452	grasses mixtures: Part-I Dry matter production, botanical composition, nitrogen

453	use efficient, nitrogen rate and yield. Asian Journal of Chemistry. 2008 Jul

454	1;20(5):4071.

455	[21] Ajdanian L, Babaei M, Aroiee H. The growth and development of cress

456	(Lepidium sativum) affected by blue and red light. Heliyon. 2019 Jul 1;5(7).

457	<https://doi.org/10.1016/j.heliyon.2019.e02109>

458	[22] Bao Y, Zhang Y, Zhang Y, Liu Y, Wang S, Dong X, Wang Y, Zhang H.

459	Screening of potential probiotic properties of Lactobacillus fermentum isolated

460	from traditional dairy products. Food control. 2010 May 1;21(5):695-701.

461	<https://doi.org/10.1016/j.foodcont.2009.10.010>

462	[23] Di Stefano V, Bongiorno D, Buzzanca C, Indelicato S, Santini A, Lucarini M,

463	Fabbrizio A, Mauro M, Vazzana M, Arizza V, Durazzo A. Fatty acids and

464	triacylglycerols profiles from Sicilian (cold pressed vs. soxhlet) grape seed oils. 465	Sustainability. 2021 Nov 25;13(23):13038. *Sustainability*, *13*(23), 13038. 466	<https://doi.org/10.3390/su132313038>

467 [[24]ISO 6557/2-1984: Fruits, vegetables and derived products - Determination of](https://www.iso.org/standard/12957.html)
468 [ascorbic acid - Part 2: Routine methods.](https://www.iso.org/standard/12957.html)
469 [25] AdebayoTayo B, Akpeji S. Probiotic viability, physicochemical and sensory
470 properties of probiotic pineapple juice. Fermentation. 2016 Dec 8;2(4):20.
471 <https://doi.org/10.3390/fermentation2040020>
472 [26] Pretscher J, Fischkal T, Branscheidt S, Jäger L, Kahl S, Schlander M, Thines E,
473 Claus H. Yeasts from different habitats and their potential as biocontrol agents.

474	Fermentation.	2018	Apr	24;4(2):31.

475	<https://doi.org/10.3390/fermentation4020031>

476	[27] Pakbin B, Razavi SH, Mahmoudi R, Gajarbeygi P. Producing probiotic peach

477	juice. Biotechnology and health sciences. 2014. [http://dx.doi.org/10.17795/bhs-](http://dx.doi.org/10.17795/bhs-24683)

478	[24683](http://dx.doi.org/10.17795/bhs-24683)

479	[28]	Pimentel TC, Madrona GS, Garcia S, Prudencio SH. Probiotic viability,

480	physicochemical characteristics and acceptability during refrigerated storage of

481	clarified apple juice supplemented with Lactobacillus paracasei ssp. paracasei

482	and oligofructose in different package type. LWT-Food science and Technology.

483	2015 Sep 1;63(1):415-22. <https://doi.org/10.1016/j.lwt.2015.03.009>

484	[29] Min M, Bunt CR, Mason SL, Bennett GN, Hussain MA. Effect of non-dairy food

485	matrices on the survival of probiotic bacteria during storage. Microorganisms.

486	2017 Aug 1;5(3):43. <https://doi.org/10.3390/microorganisms5030043>

487	[30] Kataria A, Achi SC, Halami PM. Effect of encapsulation on viability of

488	Bifidobacterium longum CFR815j and physiochemical properties of ice cream.

489	Indian	journal	of	microbiology.	2018	Jun;58:248-51.

490	<https://doi.org/10.1007/s12088-018-0720-6>

491 [31] Świeca M, Kordowska-Wiater M, Pytka M, Gawlik-Dziki U, Bochnak J, Złotek
492 U, Baraniak B. Lactobacillus plantarum 299V improves the microbiological
493 quality of legume sprouts and effectively survives in these carriers during cold
494 storage and in vitro digestion. PLoS One. 2018 Nov 21;13(11):e0207793.
495 [32] Oliveira	ME,	Garcia	EF,	Queiroga	RD,	Souza	EL.	Technological,
496 physicochemical and sensory characteristics of a Brazilian semi-hard goat
497 cheese (coalho) with added probiotic lactic acid bacteria. Scientia Agricola.

498	2012;69:370-9. <https://doi.org/10.1590/S0103-90162012000600005>

499 [33] Vinderola CG, Mocchiutti P, Reinheimer JA. Interactions among lactic acid

500	starter and probiotic bacteria used for fermented dairy products. Journal of Dairy 501	science. 2002	Apr	1;85(4):721-9. [https://doi.org/10.3168/jds.S0022-](https://doi.org/10.3168/jds.S0022-0302(02)74129-5) 502	[0302(02)74129-5](https://doi.org/10.3168/jds.S0022-0302(02)74129-5)

503	[34] Tripathi MK, Giri SK. Probiotic functional foods: Survival of probiotics during

504	processing and storage. Journal of functional foods. 2014 Jul 1;9:225-41.

505	<https://doi.org/10.1016/j.jff.2014.04.030>

506	[35] Mohan A, Hadi J, Gutierrez-Maddox N, Li Y, Leung IK, Gao Y, Shu Q, Quek

507	SY.	Sensory,	microbiological	and	physicochemical	characterisation	of

508	functional manuka honey yogurts containing probiotic Lactobacillus reuteri

509	DPC16. Foods. 2020 Jan 19;9(1):106. <https://doi.org/10.3390/foods9010106>

510 [36] Casarotti SN, Todorov SD, Penna AL. Effect of different matrices on probiotic

511	resistance to in vitro simulated gastrointestinal conditions. International journal 512	of dairy technology. 2015 Nov;68(4):595-601. [https://doi.org/10.1111/1471-](https://doi.org/10.1111/1471-0307.12215) 513	[0307.12215](https://doi.org/10.1111/1471-0307.12215)

514	[37] Chávarri M, Marañón I, Ares R, Ibáñez FC, Marzo F, del Carmen Villarán M.

515	Microencapsulation of a probiotic and prebiotic in alginate-chitosan capsules

516	improves survival in simulated gastro-intestinal conditions. International journal 517	of	food	microbiology. 2010	Aug	15;142(1-2):185-9. 518	<https://doi.org/10.1016/j.ijfoodmicro.2010.06.022>

519	[38] CHACKOSHIAN KA, Shojaosadati SA. Improvement of probiotic survival in

520	fruit juice and under gastrointestinal conditions using pectin-nanochitin-

521	nanolignocellulose	as	a	novel	prebiotic	gastrointestinal-resistant	matrix.

522	<https://doi.org/10.22037/afb.v4i3.17337>

523	[39] Popova T. Effect of probiotics in poultry for improving meat quality. Current

524	Opinion	in	Food	Science.	2017	Apr	1;14:72-7.

525	<https://doi.org/10.1016/j.cofs.2017.01.008>

526 [40] Favaro‐Trindade CS, Bernardi S, Bodini RB, De Carvalho Balieiro JC, De

527	Almeida E. Sensory acceptability and stability of probiotic microorganisms and

528	vitamin C in fermented acerola (Malpighia emarginata DC.) ice cream. Journal 529	of Food Science. 2006 Aug;71(6):S492-5. [https://doi.org/10.1111/j.1750-](https://doi.org/10.1111/j.1750-3841.2006.00100.x) 530	[3841.2006.00100.x](https://doi.org/10.1111/j.1750-3841.2006.00100.x)

531	[41] Flores-Félix JD, Silva LR, Rivera LP, Marcos-García M, García-Fraile P,

532	Martínez-Molina E, Mateos PF, Velázquez E, Andrade P, Rivas R. Plants

533	probiotics	as	a	tool	to	produce	highly	functional	fruits:	the	case	of

534	Phyllobacterium and	vitamin	C	in	strawberries.	PLoS	One.	2015	Apr

535	15;10(4):e0122281. <https://doi.org/10.1371/journal.pone.0122281>

536	[42] Ma X, Wang X, Cheng J, Nie X, Yu X, Zhao Y, Wang W. Microencapsulation

537	of Bacillus subtilis B99-2 and its biocontrol efficiency against Rhizoctonia

538	solani	in	tomato.	Biological	control.	2015	Nov	1;90:34-41.

539	<https://doi.org/10.1016/j.biocontrol.2015.05.013>

540 [43] Gerbaldo GA, Barberis C, Pascual L, Dalcero A, Barberis L. Antifungal activity

541	of two Lactobacillus strains with potential probiotic properties. FEMS 542	microbiology letters. 2012 Jul 1;332(1):27-33. [https://doi.org/10.1111/j.1574-](https://doi.org/10.1111/j.1574-6968.2012.02570.x) 543	[6968.2012.02570.x](https://doi.org/10.1111/j.1574-6968.2012.02570.x)

544 [44] Coman MM, Cecchini C, Verdenelli MC, Silvi S, Orpianesi C, Cresci A.
545 Functional foods as carriers for SYNBIO®, a probiotic bacteria combination.
546 International Journal of Food Microbiology. 2012 Jul 16;157(3):346-52.
547 [45] Afzaal M, Khan AU, Saeed F, Arshad MS, Khan MA, Saeed M, Maan AA, Khan
548 MK, Ismail Z, Ahmed A, Tufail T. Survival and stability of free and
549 encapsulated probiotic bacteria under simulated gastrointestinal conditions and
550 in	ice	cream.	Food	Science	&	Nutrition.	2020	Mar;8(3):1649.

551	<https://doi.org/10.1002/fsn3.1451>

Figure	[Click here to access/download;Figure;figures.docx](https://www2.cloud.editorialmanager.com/pone/download.aspx?id=36322816&guid=29ff221e-d045-47a6-89f1-8e882224794c&scheme=1)

**Fig 1**. **Inoculum preparation.** The final turbidity of the inoculum was equal to 0.5 McFarland and its volume was 24 ml. This was done by centrifugation of primary culture containing *Bifidobacterium bifidum (*bb12*)* and spectroscopy.

**Fig 2**. Dry matter measurement

**Fig 3. Simulated gastrointestinal conditions preparation.** The tubes from left to right, show the gastric and the intestinal conditions. MRS agar medium is used to detect the bacterial growth in these conditions.

**Fig 4**. Ascorbic acid solution, 2,6-dichlorophenol-indophenol solution and Oxalic acid are shown respectively from left to right. The Vitamin C content was determined by using these solutions and titration with 2,6-dichlorophenol-indophenol solution.

8

7

6

5.35

5

4.4

5.3

4

3.48

4.7

4.38

3

2.6

3.46

2

1.1

2.5

1

0.53

0

Control Probiotic

0.52

Day 1

0.52

0.53

0.9

Day 2

0.9

1.1

Day 3

2.5

2.6

Day 4

3.46

3.48

Day 5

4.38

4.4

Day 6

4.7

4.73

Day 7

5.3

5.35

4.73

cm

**Fig 5**. Growth rate comparison of two probiotic and control samples

a	b	c

d	e	f

**Fig 6. Plant growth process from day 1 to 6.** a: day 1 (24 hours after sowing). Seed germination. The average height of *bifidobacterium* – containing plant and the control one was respectively, 0.53 cm and 0.52 cm. b: day 2. The above vases show the *bifidobacterium* – containig plants and the below ones show the control plants. The average height of probiotic and the control plant on this day was 1.1 cm 0.9 cm respectively. c: day 3. Probiotic plant (2.6 cm), control plant (2.5 cm). d: day 4. Probiotic plant (3.48 cm), control plant (3.46 cm). e: day 5. Probiotc plant (4.4 cm), control plant (4.38 cm). f: day 6. Probiotic plant (4.73 cm), control plant (4.7 cm).

**CFU/g**

**Fig 7. Changes in the number of *Bifidobacteria* from days 0 to 3.** A decrease in the bacterial cells number is observed but the product is still a probiotic vegetable

4E+13

1.5E+13

1.5E+12

2.3E+11

1.5E+11

1.5E+10

Number of bifidobacteria

day 0

4E+13

day 3

2.3E+11

**Fig 8. Colony count result**

**Fig 9**. pH measurement of the probiotic and the control sample.

7.80

7.60

7.40

7.20

7.00

6.80

6.60

6.40

6.20

6.00

7.32

7.1

7.21

6.53

Control	Probiotic

minute 1	7.32	7.21

minute 2	7.1	6.53

**pH**

**Fig 10**. pH values of two samples at minutes 1 and 2

0.02

0.017

0.014

0.015

0.01

0.005

0

Probiotic

0.014

Control

0.017

**Acidity (%)**

**Fig 11**. The acidity difference of two samples.

**Fig 12**. Fat content difference in two samples

0.4

0.36

0.3

0.2

0.2

0.1

0

Probiotic

0.2

Control

0.36

104

102

100

98

96

94

92

90

Probiotic

Control

99.56

98.56

99.56

98.56

**Vitamin C (mg)**

**Fat (%)**

**Fig 13**. Vitamin C content of the probiotic and the control samples.

5.00

4.8	4.8	4.8

4.2

4.4

4.6

4.8

4.6	4.6

4

4.00

3.00

2.00

1.00

0.00

Aroma Taste Texture Color Appearance

Control

4.2

4.4

4.8

4.8

4.8

Probiotic

4

4.6

4.8

4.6

4.6

**Fig 14**. Sensory evaluation results as an average of five determinations.
