# Supplementary material for: Production of probiotic garden cress (Lepidium Sativum) using Bifidobacterium Bifidum and its evaluation of nutritional value, biocontrol and growth rate ability
Source: PLoS One. 2025 Jun 4;20(6):e0322552. doi: 10.1371/journal.pone.0322552 (PMC12136354; doi:10.1371/journal.pone.0322552)
Supplement: Attachment — Submitted filename: Response to Reviewers.docx [file pone.0322552.s019.docx]

Dear PLOS one team

I hope I’ve been able to amend and complete the article correctly. This is my first experience submitting an article and I was not aware of some basis needed to do so.

***Response to reviewer #1**

- ***Please follow instruction within the manuscript. authors should add the impact of research in the conclusion and introduction need more improvements to be more informative.***

The instructions have been followed and the impact of the research has been added. The introduction and the conclusion sections have been restructured and more informative.

- ***Some spelling mistakes need to be corrected***

All the spelling and numerical mistakes have been corrected.

- ***Add more details in materials section as probiotic inoculation***

All of the materials and methods have completely been added and the probiotic inoculation method has also been rewritten and fully explained.

***Response to reviewer #2**

- ***Where is the key word?***

The key words are added to the end of the abstract section

- ***SGI must be written fully for the first time in abstract.***

SGI is fully rewritten in the abstract.

- ***Line 93 SGI can be written in abbreviated form as it is mentioned before.***
- ***Line 123 correct the word moistens.***
- ***Line 175 they were determined instead of and were determined.***

All the spelling, abbreviation and grammatical mistakes have been corrected.

- ***Why the authors didn’t make different intervals in determination of total viable count for studying the effect of stomach condition, also why they didn’t make different PH values?***
- ***Why the authors didn’t make different intervals in determination of total viable count for studying the effect of SGI with different concentration for bile condition?***

The strain used in this study (*Bifidobacterium bifidum*) is an industrial strain whose viability and resistance to gastrointestinal condition and pH has already been examined and confirmed. We aimed to investigate the ability of the bacteria to survive in stomach and intestinal conditions due to the change in bacterial niche (The original niche of this strain is the human large intestine not the herbs). For this reason, only the pH mentioned in the references and articles discussing probiotics viability was used to check the survival of the bacteria and there was no need to use different concentrations of bile salts or other pH values.

Determination of total viable count after exposure to SGI condition was done on day 3 because it was important for us that the bacteria survive in the mentioned conditions until the last day of expiration. That is why different intervals was not made.

- ***Line 220 write the name of PDA media (Potato Dextrose Agar) media fully.***

The full form of PDA media is written.

- ***Statistical Analysis is required.***

Some tests including fat content, pH and acidity measurement was repeated two more times with the mentioned protocols to add statistical analysis you asked for. The other parameters, except organoleptic evaluation, are reported as an average of three results obtained from previous experiments but the statistical analysis is also added to them, hoping that it will be acceptable to you.

- ***Conclusion: The whole conclusion should be summarized and include recommendation with mentioning the most important finding not to repeat all the result that presented in detail in the result section.***

The most important findings and recommendations are added to the conclusion section and the results repetition is removed.

***Response to reviewer #3**

***- The manuscript needs English editing, reconstitution of sentences***

 English editing and reconstitution of sentences are done. I tried to rewrite the manuscript in an intelligible fashion and standard English.

- ***Follow Reviewer comments***

All the comments have been applied and followed.

- ***No statistical analysis was performed***

The statistical analysis is performed and added.

- ***Follow journal guidelines***

The journal guidelines have been followed.

- ***Have the authors made all data underlying the findings in their manuscript fully available?***

Yes; The authors made all data underlying the findings in the manuscript fully available without restriction.

All the mistakes the dear reviewers have mentioned were completely correct and acceptable and I am very grateful that you read my article carefully.

Thank you for giving me another chance to send you the revised manuscript. I hope I can send you more articles and have more cooperation with you.

Kind regards

Golnaz Shambayati
