# Supplementary material for: Production of probiotic garden cress (Lepidium Sativum) using Bifidobacterium Bifidum and its evaluation of nutritional value, biocontrol and growth rate ability
Source: PLoS One. 2025 Jun 4;20(6):e0322552. doi: 10.1371/journal.pone.0322552 (PMC12136354; doi:10.1371/journal.pone.0322552)
Supplement: Attachment — Submitted filename: Response_to_Reviewers_auresp_3.docx [file pone.0322552.s021.docx]

Dear PLOS one team

I hope I’ve been able to amend and complete the article correctly. This is my first experience submitting an article and I was not aware of some basis needed to do so.

***Response to reviewer #1**

- ***Please follow instruction within the manuscript. authors should add the impact of research in the conclusion and introduction need more improvements to be more informative.***

The instructions have been followed and the impact of the research has been added. The introduction and the conclusion sections have been restructured and more informative.

- ***Some spelling mistakes need to be corrected***

All the spelling and numerical mistakes have been corrected.

- ***Add more details in materials section as probiotic inoculation***

All of the materials and methods have completely been added and the probiotic inoculation method has also been rewritten and fully explained.

***Response to reviewer #2**

- ***Where is the key word?***

The key words are added to the end of the abstract section

- ***SGI must be written fully for the first time in abstract.***

SGI is fully rewritten in the abstract.

- ***Line 93 SGI can be written in abbreviated form as it is mentioned before.***
- ***Line 123 correct the word moistens.***
- ***Line 175 they were determined instead of and were determined.***

All the spelling, abbreviation and grammatical mistakes have been corrected.

- ***Why the authors didn’t make different intervals in determination of total viable count for studying the effect of stomach condition, also why they didn’t make different PH values?***
- ***Why the authors didn’t make different intervals in determination of total viable count for studying the effect of SGI with different concentration for bile condition?***

The strain used in this study (*Bifidobacterium bifidum*) is an industrial strain whose viability and resistance to gastrointestinal condition and pH has already been examined and confirmed. We aimed to investigate the ability of the bacteria to survive in stomach and intestinal conditions due to the change in bacterial niche (The original niche of this strain is the human large intestine not the herbs). For this reason, only the pH mentioned in the references and articles discussing probiotics viability was used to check the survival of the bacteria and there was no need to use different concentrations of bile salts or other pH values.

Determination of total viable count after exposure to SGI condition was done on day 3 because it was important for us that the bacteria survive in the mentioned conditions until the last day of expiration. That is why different intervals was not made.

- ***Line 220 write the name of PDA media (Potato Dextrose Agar) media fully.***

The full form of PDA media is written.

- ***Statistical Analysis is required.***

Some tests including fat content, pH and acidity measurement was repeated two more times with the mentioned protocols in order to add statistical analysis you asked for. The other parameters, except organoleptic evaluation, are reported as an average of three results obtained from previous experiments but the statistical analysis is also added to them, hoping that it will be acceptable to you.

- ***Conclusion: The whole conclusion should be summarized and include recommendation with mentioning the most important finding not to repeat all the result that presented in detail in the result section.***

The most important findings and recommendations are added to the conclusion section and the results repetition is removed.

***Response to reviewer #3**

***- The manuscript needs English editing, reconstitution of sentences***

 English editing and reconstitution of sentences are done. I tried to rewrite the manuscript in an intelligible fashion and standard English.

- ***Follow Reviewer comments***

All the comments have been applied and followed.

- ***No statistical analysis was performed***

The statistical analysis is performed and added.

- ***Follow journal guidelines***

The journal guidelines have been followed.

- ***Have the authors made all data underlying the findings in their manuscript fully available?***

Yes; The authors made all data underlying the findings in the manuscript fully available without restriction.

***Response to reviewer #4:**

- ***The Introduction is well written, but the length is a bit short. Please add some content and references appropriately***

The introduction is written longer and the content related to this section is added properly.

- ***The vertical axis of Figure 4 needs to be explained.***

Done!

- ***There are too many images in the manuscript, so it is necessary to consider combining them into larger images, or some images can be included in the appendix. Especially the merging of Figures 11-13.***

You are completely right. I felt the same when looking at the file. Some unnecessary figures have been eliminated and the figures and tables labels are also changed due to the rearranges in the figures and tables order.

- ***Similarly, the results section is also appropriately merged together based on the merging of the graphs.***

Done!

- ***The conciseness of the research conclusions is not enough, for example, the conclusion and abstract sections do not have a research significance section, only the research results***

No doubt, the points you mentioned are definitely right. Thank you for your points of view. I should have mentioned the importance of my research and highlight it in order the readers know WHY I decided to go work on this topic. I tried my best to show its importance in the conclusion and abstract section. I hope I was successful.

- ***Please include the following items when submitting your revised manuscript:***
- ***A rebuttal letter that responds to each point raised by the academic editor and reviewer(s). You should upload this letter as a separate file labeled 'Response to Reviewers'.***
- ***A marked-up copy of your manuscript that highlights changes made to the original version. You should upload this as a separate file labeled 'Revised Manuscript with Track Changes'.***
- ***An unmarked version of your revised paper without tracked changes. You should upload this as a separate file labeled 'Manuscript'.***

The ‘*Response to reviewers*’ and ‘*Manuscript’* files have already been written and prepared. But unfortunately I had a problem preparing ‘*Revised Manuscript with Track Changes*’ file. I did not get your mean by “Track Changes” and every time you mentioned a mistake and asked for a revision, I highlighted the edited section to let you know that this part have been edited, eliminated or added to the manuscript. Actually I have included the changes in the highlighted sections of the document. I am so sorry for that. I have been dealing with many problems lately and it completely confused me. It would be a great favor if you accept this version of my manuscript. Thank you. I’ll be sending you more papers soon and I promise I will never ever do this mistake again.

All the mistakes the dear reviewers have mentioned were completely correct and acceptable and I am very grateful that you read my article carefully.

Thank you for giving me another chance to send you the revised manuscript. I hope I can send you more articles and have more cooperation with you.

Kind regards

Golnaz Shambayati
